# Current advances and potential trends of the polysaccharides derived from medicinal mushrooms sanghuang

**DOI:** 10.3389/fmicb.2022.965934

**Published:** 2022-08-03

**Authors:** Hao Wang, Jin-Xin Ma, Miao Zhou, Jing Si, Bao-Kai Cui

**Affiliations:** Institute of Microbiology, School of Ecology and Nature Conservation, Beijing Forestry University, Beijing, China

**Keywords:** sanghuang, polysaccharide, preparation strategy, structural characterization, biological activity

## Abstract

For thousands of years, sanghuang is distinctive as a general designation for a group of precious and rare Chinese medicinal mushrooms. Numerous investigations have revealed that polysaccharide is one of the important biological active ingredients of sanghuang with various excellent biological activities, including antioxidant, anti-aging, anti-tumor, immunomodulatory, anti-inflammatory, anti-diabetic, hepatoprotective, and anti-microbial functionalities. For the past two decades, preparation, structural characterization, and reliable bioactivities of the polysaccharides from fruiting bodies, cultured mycelia, and fermentation broth of sanghuang have been arousing extensive interest, and particularly, different strains, sources, and isolation protocols might result in obvious discrepancies in structural features and bioactivities. Therefore, this review summarizes the recent reports on preparation strategies, structural features, bioactivities, and structure-activity relationships of sanghuang polysaccharides, which will enrich the knowledge on the values of natural sanghuang polysaccharides and support their further development and utilization as therapeutic agents, vaccines, and functional foods in tonic and clinical treatment.

## Introduction

Sanghuang, also called as “sang'er,” “sangchen,” and “sanghuanggu,” was first recorded in the medicinal utilization dating back to 2000 years in China's Shennong's Herbal Classic of Materia Medica. Compendium of Materia Medica written by Shi-Zhen Li in the Ming Dynasty also clearly described its medicinal effects (Wu, 2012; Zhu and Cui, 2016). Since then, sanghuang has been around and consumed as a food and versatile medicine in Chinese traditional medicine due to its excellent activities to treat dysentery, metrorrhagia, amenorrhea, aging, poisoning, and digestion (Wu, 2012; Hsieh et al., 2013; Zhang et al., 2016). Until 1968, modern pharmacological investigation on sanghuang was unlocked based on the finding of its remarkable anti-tumor activity by Ikekawa et al. (1968). As a group of ancient, precious, natural, and medicinal fungal resources, sanghuang has been validated by extensive scholars to have fascinating therapeutic functionalities, such as anti-tumor (Huang et al., 2011; He et al., 2021a), antioxidant (Liu et al., 1997; Shon et al., 2003; Chen et al., 2016), anti-bacterial (Lu et al., 2020; Ma et al., 2021), anti-virus (Mirzadeh et al., 2020), anti-angiogenic (Lee Y. S. et al., 2010), anti-platelet (Kamruzzaman et al., 2011), anti-inflammatory (Kim B. C. et al., 2006; Lin et al., 2017), immunoregulatory (Meng et al., 2016; Azeem et al., 2018; Jung and Kang, 2020), and hepatoprotective activities (Shan et al., 2019; Yang et al., 2020), treating diabetes (Hwang et al., 2005; Ajith and Janardhanan, 2021), hyperlipidemia (Rony et al., 2014), and cardiovascular (Lee et al., 2007; Su et al., 2017) diseases, etc.

Polysaccharides, which act as “biological response modifiers,” are macromolecules with long complex chains made of aldose or ketose joined by glycosidic bonds and are involved in many biological processes (Leung et al., 2006; Barbosa and de Carvalho Junior, 2020). Currently, the polysaccharides extracted from natural resources have drawn widespread concern around the world for their safety, non-toxic, and, thus, potential applications in functional food and biomedical products (Singdevsachan et al., 2016; Liu L. Q. et al., 2019; Yang M. Y. et al., 2019; Wang H. et al., 2021). Sanghuang has long been recognized as a class of famous medicinal mushrooms and contains a huge variety of biologically active ingredients, including polysaccharides (Yang et al., 2020), flavonoids (Wu et al., 2019) triterpenoids (Rajachan et al., 2020), polyphenols (Hwang et al., 2015; Liu et al., 2017), alkaloids (Wu et al., 2019), enzymes (Wang et al., 2020a,b), lipids (He et al., 2021b), and other compounds (Chen et al., 2016; Martinez-Medina et al., 2021), which have been manifested to possess various biological activities (Aida et al., 2009; Reis et al., 2017; Wen et al., 2019; Shi et al., 2020; Yang et al., 2020; Ajith and Janardhanan, 2021). Especially, the most dominant active component of sanghuang is a polysaccharide, which exerts various health-promoting effects, including free-radical scavenging ability (Gong et al., 2020), anti-tumor (Chakraborty et al., 2019), anti-microbial (He et al., 2021b), anti-inflammatory (Hou et al., 2020), neuroprotective (Chen et al., 2019), hepatoprotective (Yuan et al., 2019), anti-diabetic (Gong et al., 2020), and immunomodulatory (Meng et al., 2016) activities. An ever-growing number of structurally multifarious polysaccharides have been prepared from the fruiting bodies, cultured mycelia, and fermentation broth of sanghuang, by large quantities of extraction and isolation methods, such as hot water extraction, and ultrasonic- and enzymatic-assisted extraction, as well as substantial purification protocols, such as ethanol precipitation and column chromatography filled with different packings (Zhang Y. et al., 2015; Ren et al., 2019; Liu Y. H. et al., 2020; Mirzadeh et al., 2020; Leong et al., 2021). It is worth noting that the varied sources of original strains and preparation strategies can result in differences in the structures and properties of polysaccharides. Moreover, the bioactivities of polysaccharides are speculated to be significantly affected by their chemical structures, including molecular weights (Mws), monosaccharide compositions, uronic acid contents, primary structures, water solubilities, spatial configurations, and positions and types of glycosidic linkages and branches, sulfates contents, polymer charges, etc. (Villares et al., 2012; Meng et al., 2016; Yan et al., 2017; Wang W. J. et al., 2020; He et al., 2021b), but the accurate structure-bioactivity relationships are still not quite clear.

**Figure 1:**
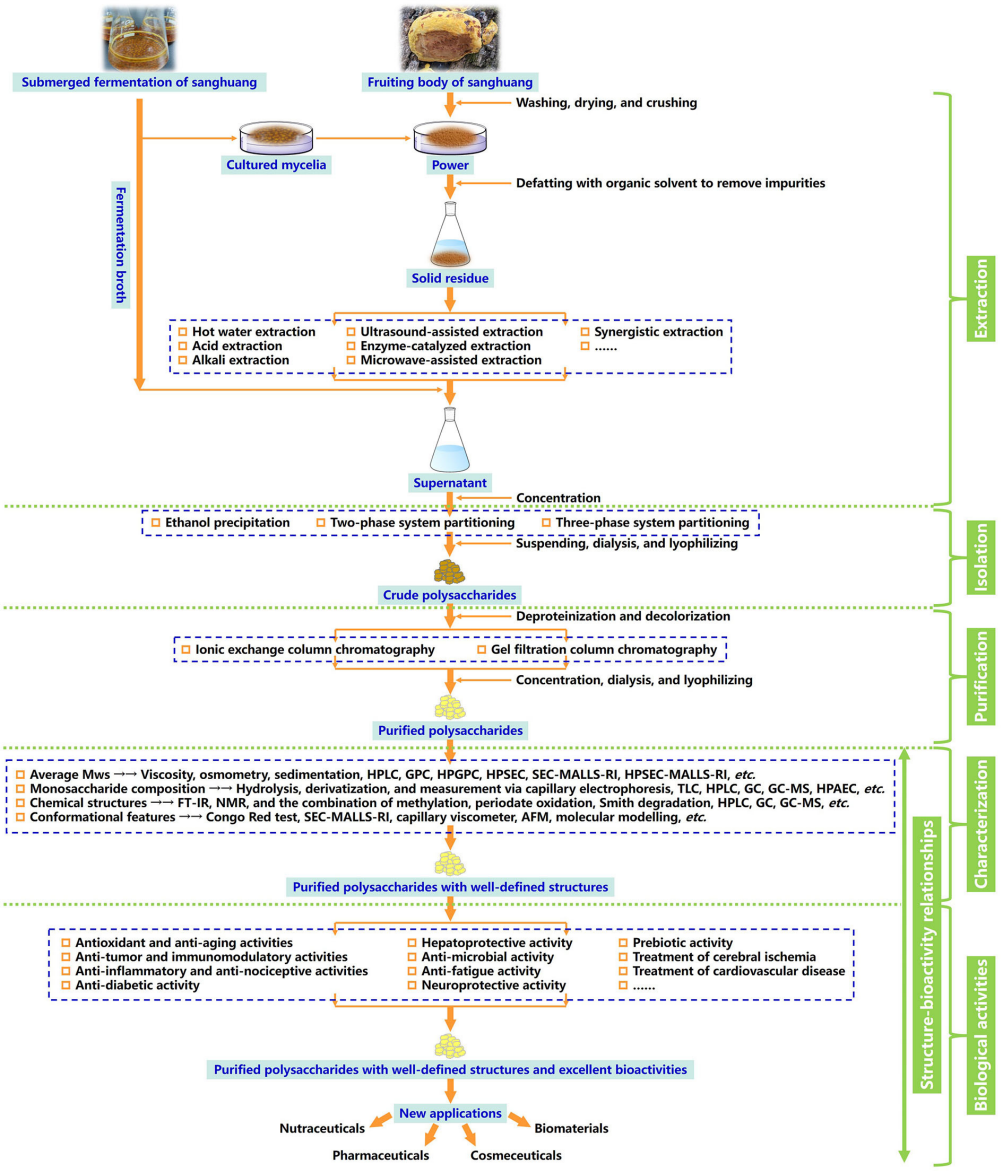
Schematic illustration of preparation, structural elucidation, and bioactivity determination of the polysaccharides derived from the naturally medicinal fungal resources sanghuang.

**Figure 2:**
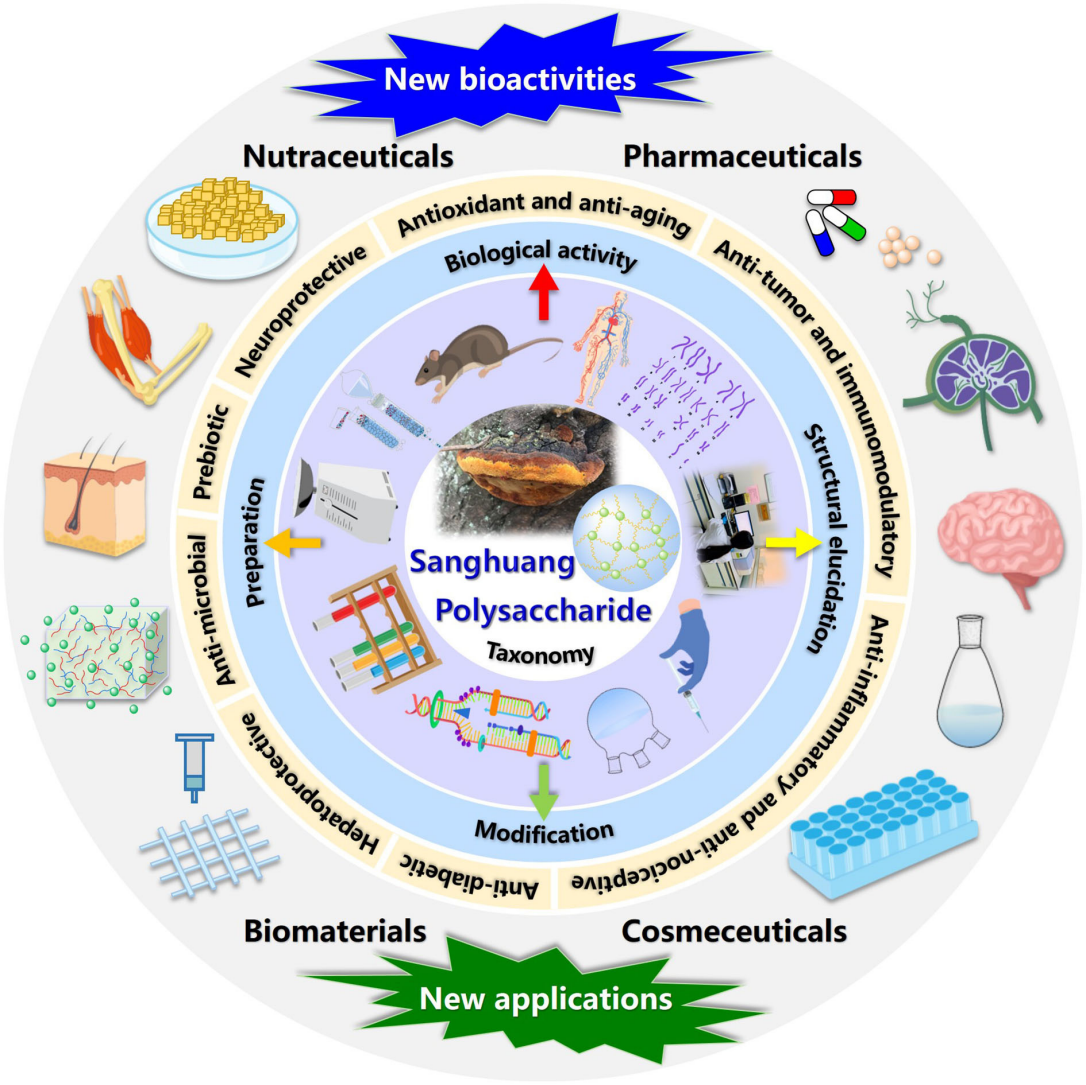
The overall highlights discussed in this review.

In this review, the evolution of sanghuang species and their names, preparation strategies, and emerging techniques of structural characterization, as well as biological activities detected of a series of overcovered bioactive polysaccharides from the fruiting bodies, cultured mycelia, and fermentation broth of sanghuang in recent years, were systematically summarized. In addition, the relationships between structural features and biological activities were discussed, and the future development trends of sanghuang polysaccharides were also prospected, aiming at benefiting the comprehensive understanding of the polysaccharides from natural medicinal fungal resources and providing valuable guidance for future research and applications on sanghuang polysaccharides-based therapeutic drugs, vaccines, and health-promoting functional foods in tonic and clinical treatment. The schematic illustration of preparation, structural elucidation, and bioactivity determination of sanghuang polysaccharides was demonstrated in Figure 1. The overall highlights of this review were visualized in Figure 2.

Herein, the sanghuang polysaccharides have been reported in recent years and the detailed information regarding their strains, sources, extraction, isolation and purification methods, Mws, monosaccharide compositions, structural features, bioactivities, involved mechanisms, and structure-bioactivity relationships, as well as their references, were integrated into Supplementary Tables 1, 2.

## Evolution of species and their names

There was much controversy and confusion concerning the sanghuang species and their accurate scientific names in the past. The term *Phellinus linteus* (Berk. & M.A. Curtis) Teng had been universally believed as the scientific name for sanghuang (Teng, 1939). Dai and Xu (1998) found that *P. linteus* was a species of Central America, not Asia. The species distributed in China, Japan, and Korea was considered to be *P. baumii* Pilát. During this period, some scholars supported that the scientific name of sanghuang was *P. igniarius* (L.) Quél. (Liu, 1978). Wu et al. (2012) published the authentic sanghuang as a new species, *Inonotus sanghuang* Sheng H. Wu, T. Hatt. & Y.C. Dai that only grows on *Morus*. Then, Zhou et al. (2016) proposed the new genus *Sanghuangporus* Sheng H. Wu, L.W. Zhou & Y.C. Dai to amend *I. sanghuang* and its closely related species, and *S. sanghuang* (Sheng H. Wu, T. Hatt. & Y.C. Dai) Sheng H. Wu, L.W. Zhou & Y.C. Dai had become the scientific name for the sanghuang species growing on *Morus*. Wu et al. (2020) found a new species, *S. vitexicola*, from tropical Taiwan, China. In 2021, a new species, *S. subbaumii* Shan Shen, Y.C. Dai & L.W. Zhou, belonging to this genus had been discovered by Shen et al. (2021). To date, the genus *Sanghuangporus* consists of 15 species, which are widely distributed across cold temperate to subtropical and tropical zones. However, some species only appeared in specific regions and form a strict parasitic relationship with their host tree species; for example, *S. sanghuang* only grows on *Morus* (Zhu et al., 2019). Some researchers also endorsed that the sanghuang recorded in ancient Materia Medica might be *I. hispidus* (Bull.) P. Karst. (Bao et al., 2013). Consequently, the generalized sanghuang comprises the genera *Sanghuangporus, Phellinus, Inonotus*, and other species in the family Hymenochaetaceae, characterized by their yellow-brown and hard fruiting bodies (Wu and Dai, 2020).

## Extraction, isolation, and purification

The extraction, isolation, and purification of polysaccharides are fundamental to further research on their monosaccharide compositions, Mws, primary structures, types and degrees of branching, configurations, bioactivities, and structure-activity relationships (Zhang et al., 2007; Ferreira et al., 2015; He et al., 2021b; Maity et al., 2021). To effectively improve the purity, the fruiting bodies, cultured mycelia, or fermentation broth are usually amenable to an array of extraction, isolation, and purification steps, such as defatting with an organic solvent to remove lipids and other impurities, extraction using hot water, acid, alkali, or other assisted treatment, subsequent fractional precipitation by ethanol with various concentrations, deproteination with a Sevag reagent or protease enzymolysis, decolorization by resin or activated carbon, dialysis, concentration, lyophilization, and final refinement through ionic exchange column chromatography, gel filtration chromatography, affinity chromatography, etc. (Shi, 2016; Ren et al., 2019; Shi et al., 2020; Huang et al., 2021). Nevertheless, the obstacles to the acquirement of the purified functional polysaccharides from sanghuang is the structural changes and loss in activity caused by unsuitable approaches and operating conditions. It is essential to seek the appropriate schemes for extraction, isolation, and purification of these biological macromolecules according to their characteristics, to ensure that sanghuang polysaccharides can be commercialized to a large extent.

Fungal polysaccharides can be classified by their locations in cells, namely, intracellular polysaccharides from the fruiting bodies and cultured mycelia and exopolysaccharides from the fermentation broth. Generally, most exopolysaccharides are water soluble, and can be directly extracted by the water, acidic, or alkaline solutions (Cheng et al., 2018; Tang et al., 2020; Leong et al., 2021; Wu et al., 2022a). Hot water extraction as the most classical, convenient, and common extraction method should be operated using water at ≥50°C for a long duration of 1.5–10 h. Yan et al. (2016b) extracted an antioxidant activity-containing polysaccharide from the *P. linteus* mycelia through immersing the defatted mycelia into distilled water at 95°C for 8 h, then extracted twice. Similar extraction protocols were deployed by Ge et al. (2009) for gaining a water-soluble polysaccharide from the fruiting bodies of sanghuang mushroom *P. baumii* using hot water extraction (extracted thrice for 2 h each at 100°C). However, this extraction approach has several drawbacks containing the high reaction temperature, large material consumption, prolonged extraction time, etc. (Kang et al., 2019). Extraction conditions, including temperature, time, and the ratio of raw material to solvent are the important factors influencing the extraction yield of sanghuang polysaccharides (Huang et al., 2012; Tang et al., 2020). Wang et al. (2014a) extracted polysaccharides from the *P. nigricans* mycelia and verified the effects of time, temperature, frequency, and the ratio of water to raw material *via* central composite design in response surface methodology. The optimal conditions were extraction time of 2.8 h, the ratio of water to the raw material of 28, frequency of 5, and the temperature of 95°C. Under these conditions, the maximal yield of crude polysaccharides was 15.33 ± 0.21%. An increase in extraction temperature can accelerate the extraction yield of polysaccharides from sanghuang into the water to a certain level, followed by possible loss, due to the thermal degradation and structural modification at an excessive temperature, even resulting in decreases in Mws, as evidenced by Guo et al. (2010) and Zheng et al. (2019). A longer extraction time also usually shows a promotion in extraction yield (Guo et al., 2010). Li et al. (2019) found that an excessively lower ratio of water to raw material may lead to enhanced viscosity and incomplete extraction, while the comparatively higher ratio may incur an increased difficulty of further separation and purification, impacting the overall processing time, costs, and resources. Therefore, water-material ratio acts a key role in the hot water extraction of polysaccharides.

In acid or alkali extraction, acids such as HCl or (NH_4_)_2_C_2_O_4_, and alkalis such as NaOH or NaBH_4_, are devoted to drive the release of polysaccharides from sanghuang (Zhang et al., 2007; Yan et al., 2017; Tang et al., 2020). Wang Z. B. et al. (2014) adopted the successive extraction, including hot water, 1% (NH_4_)_2_C_2_O_4_, and 1.25 M NaOH/.05% NaBH_4_ to get *P. linteus* polysaccharides. The alkali extract with a high Mw and the highest carbohydrate and uronic acid contents exhibited stronger bioactivities when compared with other extracts. Wang K. et al. (2020) also employed 1.25 M NaOH/.05% NaBH_4_ to extract polysaccharides from the *P. linteus* mycelia, and the resulting fraction composed of Ara, Xyl, Glc, and Gal with the molar ratio of 5.5:7.8:1.8:1 exerted radical scavenging capacities and antioxidant activities *in vitro* and *in vivo*. The reason for the acid or alkali extraction often brings out the elevated extraction yields of polysaccharides is that destruction of cell walls of fruiting bodies and cultured mycelia of sanghuang and the degradation of fiber structures by acid or alkali solution, hence, allowing the release of more intracellular polysaccharides and transformation of water-insoluble into water-soluble fractions (Zhang Y. et al., 2015).

As fruiting bodies and cultured mycelia are incompletely water-soluble, other alternatives, such as ultrasound, enzyme, and microwave, which can provoke the breakdown of the cell walls of mushrooms and improve the solubility of polysaccharides, are introduced to assist in harvesting the higher yield of polysaccharides (Ebringerová and Hromádková, 2010; Cheng et al., 2012; Marić et al., 2018; Shi et al., 2019; Chang et al., 2021; Wu et al., 2022b). Zhang et al. (2014) established a flat-plate ultrasound technique to stimulate the production of the polysaccharide from the *P. igniarius* mycelial fermentation. Optimal conditions obtained by a three-factor-three-level Box-Behnken design were ultrasound treatment time of 65 min, duty cycle time of 25 s, and culture time of 3.8 d that gave a maximal polysaccharide yield of 1.8002 g/L, which increased by ~22.64%, compared with the treatment without ultrasonication. LSCM observations revealed that ultrasound can change the morphology and structure and of fungal mycelia, and then, accelerate the mass transfer of metabolites. Also, Liu et al. (2019a) inspected that the yield of *I. hispidus* polysaccharide with ultrasonic-assisted extraction was 39% higher than that with the non-ultrasound control group, suggesting that ultrasound-assisted extraction is a more effective method for polysaccharide extraction using its cavitation generated by rapid formation, implosion of the tremendous bubbles in mobile phase, and release of a vast of hydrodynamic forces, thus, driving the cell wall disruption, mass transfer, osmotic force, etc. (You et al., 2014; Chen X. H. et al., 2021; Wu et al., 2022b). Efficient degradation of the cell walls of mushrooms can be achieved by enzymes, producing more polysaccharides from the inside cells, whose extraction rates depend on the type and amounts of enzymes, temperature, pH, the ratio of liquid to solid material, reaction time, etc. (Marić et al., 2018; Cui and Zhu, 2021). A new procedure for the enzyme-catalyzed extraction of polysaccharides from *P. igniarius* was developed by Xu et al. (2016). Based on the single-factor experiment and Box-Behnken design, the optimum technological conditions were pH 5.79, reaction time of 1.5 h, extraction temperature of 55.02°C, and enzyme dosage of 3.04%, respectively. Under these conditions, the extraction rate of polysaccharides was increased by 49.5% compared with conventional hot water extraction. However, the higher costs of enzymes and sensitivity to harsh surroundings are the main shortcomings of enzyme-assisted extraction (Marić et al., 2018). Microwave is a radio wave with the frequency range at 300–3 × 10^5^ MHz, which appears *via* interactions between polar molecules and is influenced by the external pressure, temperature, and time, whereupon being advocated to be a feasible tool for extraction of polysaccharide macromolecules (Singh et al., 2012; Mirzadeh et al., 2020). Gao et al. (2017) extracted the *P. igniarius* polysaccharides from six different origins using the microwave extraction method, and the resulting Gansu polysaccharide was identified to have a great potential as a natural anti-tumor agent with immunoregulatory activities. Furthermore, synergistic treatments for incorporating the superiority of these extraction methods can serve as the other beneficial choices in extracting active substances from sanghuang (Shi, 2016; Leong et al., 2021). For example, Ying et al. (2019) optimized the extraction procedure of polysaccharides from the fruiting bodies of *P. igniarius* by using the ultrasonic-microwave synergistic approach. Rank order from the highest to the lowest effects of the three extraction factors on the polysaccharides yields was ultrasonic power > extraction time > microwave power. The consequences displayed that the polysaccharides extracted by the synergistic method performed higher antioxidant and anti-tumor activities, which implied that the combination of ultrasound and microwave comes as a time-saving and high-yield method for polysaccharides assisted extraction.

Subsequently, the aqueous extracts or fermentation broth of sanghuang can be subjected to ethanol precipitation with ~95% (V/V) ethanol, in addition to fractional precipitation with various gradients of ethanol or acid precipitation with acetate acid, to isolate the crude polysaccharides mixed with proteins and pigments. Aqueous two-phase system and three-phase partitioning as alternative tools referring to the sequential mixing of organic solvent and salt with crude extracts or suspensions to reach the different dispense phases have been exploited for the efficient extraction and separation of polysaccharides as well (Chew et al., 2019; Wang et al., 2019b; Wu Y. et al., 2022). Wang et al. (2019a) engaged in three-phase partitioning to directly isolate a bioactive exopolysaccharide from the cultured broth of *P. baumii* and gave the maximal extraction yield of 52.09% under the following conditions: 20% (w/v) (NH_4_)_2_SO_4_ concentration, 1.0:1.5 (V/V) ratio of broth to *t*-butanol, 30 min, and 35°C. This polysaccharide with high carbohydrate and uronic acid contents demonstrated dramatically radical-scavenging, antioxidant, α-amylase and α-glycosidase inhibitory, as well as macrophage-stimulating activities.

To further eliminate the biological wastes, and then acquire the homogenized samples, the crude polysaccharides should go through a set of adsorbents and eluents, including chemical/enzymatic deproteinization, decolorization by resin or activated carbon, dialysis to remove small molecules, concentration, and freeze-drying, followed by ionic exchange column chromatography (DEAE-Cellulose, DEAE-Sepharose, DEAE-Sephadex, etc.) to seize the neutral and acidic polysaccharides, and gel filtration column chromatography (Sepharose, Sephadex, Sephacryl, etc.) to capture the polysaccharides with different Mws, eluting with suitable eluates and speeds, collecting, dialyzing, condensing, and lyophilizing (Fang and Ding, 2007; Tang et al., 2020; Leong et al., 2021). During the entire steps, the associated carbohydrate and protein concentrations of each polysaccharide fraction were measured *via* the phenol-sulfuric acid method with Glc as a standard (DuBois et al., 1956) and the Bradford method with bovine serum albumin as a standard (Bradford, 1976). Yang et al. (2007) utilizes the hot water extraction, ethanol precipitation, DEAE-Sepharose Fast-Flow, and High-Resolution Sephacryl S-400 column chromatography to isolate a new *P. igniarius* fruiting bodies-derived heteropolysaccharide, whose structural investigation was carried out with HPLC, HPAEC, GC-MS, methylation analysis, and NMR spectroscopy techniques. Xue et al. (2011) separated a 17-kDa water-soluble polysaccharide from the *P. baumii* mycelia taking advantage of the hot water extraction, ethanol precipitation, and DEAE-Sephadex A-50 and LPLC-Sephadex G-75 chromatography methods, as well as confirming that this polysaccharide owned immunomodulatory and anti-tumor activities *in vitro*. Two different polysaccharides from the submerged mycelia culture of *P. mori* were the ethanol precipitated and gel filtration chromatographed by Cao et al. (2013). Besides, they substantiated that the polysaccharides had (1 z → 4)-linked Man*p* backbones, with branches of (1 → 4)-linked glucosyl residues and (1 → 3,4)-linked Gal*p* residues. Cheng et al. (2020a) fractionated a novel exopolysaccharide from the liquid culture broth of *S. sanghuang* by using ethanol precipitation, DEAE-Sepharose Fast Flow, and Sephacryl S-100 HR gel column chromatography. This exopolysaccharide, exclusively composed of Man, consisted of 1,3-linked and 1,2-linked α-_D_-Man*p* with substitution at *O*-6 of 1,2-linked α-_D_-Man*p* by 1,6-linked α-_D_-Man*p* residues and terminal α-_D_-Man*p* residues, and exerted potential anti-tumor activity against the growth of HepG2 and MCF7 *in vitro*.

## Physicochemical and structural properties

Up to present, the versatile natural polysaccharides derived from sanghuang have been prepared and structurally characterized, as listed in Supplementary Table 1, because of their plentiful promising bioactivities (Zong et al., 2012; Meng et al., 2016; Ren et al., 2019; Gong et al., 2020). It is widely accepted that the functions and behaviors of these complex biological macromolecules are dramatically affected by their physicochemical and structural properties, which are involved in composition and structural uniqueness, mainly covering Mws, sequences of monosaccharide residues, configurations and conformation of isomers, types and numbers of glycosidic linkages, skeleton lengths, chain compositions and aggregations, presences and positions of branches and functional groups, branching degrees, lengths of side chains, 3D conformation, etc. (Ferreira et al., 2015; Ruthes et al., 2016; Wang J. Q. et al., 2016; Xie et al., 2020). Although there exist some restrictions for accessing the fine structures in all hierarchies due to the structural diversity and variability of macromolecules, the basic chemical structures of purified sanghuang polysaccharides have been probed and identified by combined means of the chemical, instrumental, and biological techniques, including hydrolysis (Cao et al., 2019), methylation analysis (Sun et al., 2021), periodate oxidation (Nypelö et al., 2021), Smith degradation (Villares et al., 2012), Congo Red test (Guo X. Y. et al., 2021), capillary viscometer (Yuan et al., 2018), Zeta-sizer Nano instrument (Miao et al., 2020), UV-visible spectrophotometry (Guo X. Y. et al., 2021), HPLC (Yang et al., 2007), HPSEC (Wang et al., 2014b), HPSEC-MALLS-RI (Cheong et al., 2015), HPGPC (Ren et al., 2019), TLC (Yang P. et al., 2019), GC (Lo et al., 2011), MS (Liu et al., 2021), GC-MS (Ma et al., 2016), HPAEC (Zhang Z. F. et al., 2015), FT-IR (Qian et al., 2009), NMR spectroscopy (^1^H, ^13^C, COSY, DEPT, HMBC, HMQC, HSQC, NOESY, and TOCSY) (Doost et al., 2019; Yao et al., 2021), XRD (Qian et al., 2009; Guo X. Y. et al., 2021), DLS (Wang Z. B. et al., 2014), DSC (Guo X. Y. et al., 2021), TGA (Guo X. Y. et al., 2021), AFM (Guo X. Y. et al., 2021), enzymatic analysis (Karaki et al., 2016; Mitchell et al., 2021), immune analysis (Sharma et al., 2019), etc. Recently, some emerging methods have also been applied for the structural characterization of polysaccharides (Fabijanić et al., 2019; Li Y. T. et al., 2020; Nandita et al., 2021).

### Average molecular weight

Polysaccharides, as a structurally diverse class of biological macromolecules, commonly possess the relatively higher Mws, which have been determined by viscosity measurement (Yan et al., 2016a), osmometry (Gong et al., 2020), sedimentation (Ren et al., 2019), HPLC (Jiang et al., 2015), GPC (Wu et al., 2013), HPGPC (Zhang et al., 2018; Wu Y. et al., 2022), HPSEC (Lee S. M. et al., 2010), SEC-MALLS-RI (Jia et al., 2017; Cheng et al., 2020a), HPSEC-MALLS-RI (Li et al., 2015; Wang et al., 2019b), etc. Pei et al. (2015) used HPGPC to estimate the average Mw of a polysaccharide prepared from the alkaline extract of the *P. linteus* mycelia as 3.43 × 10^5^ kDa. Li et al. (2015), by virtue of HPSEC-MALLS-RI, unearthed that the Mws of nine polysaccharides from the fruiting bodies, submerged mycelia, and solid-state-fermented products of *P. baumii*, decreased with the increasing precipitated ethanol concentrations. Mws of the polysaccharides from the fruiting bodies and submerged mycelia ranged from 19.8 to 1.89 × 10^3^ kDa and 2.11 × 10^3^ to 2.01 × 10^4^ kDa, respectively. Some lower-Mw fractions were detected in the solid fermented products. As summarized in Supplementary Table 1, the polysaccharides derived from various sanghuang species and experimental conditions exhibit wide Mws ranging from 1 to 1 × 10^6^ kDa.

A correlation between the antioxidative potency and Mw of polysaccharides had been well-established according to the substantial research (Wang J. Q. et al., 2016; Yan et al., 2016a; Gong et al., 2020; Wu Y. et al., 2022). It is pronounced that the antioxidation and free radicals scavenging of low-Mw polysaccharides are superior to that of the polysaccharides with high Mws, on account of more active intramolecular hydrogen bonding effect of O–H and electron-donating substituents (Xing et al., 2005). Wu Y. et al. (2022) separated a low-Mw polysaccharide from the *P. linteus* mycelia by using an aqueous two-phase system based on choline chloride ([Chol]Cl)/K_2_PO_4_, which owned more prominent scavenging effect for radicals when compared to the higher-Mw polysaccharide obtained using the ethanol precipitation and isolation protocols. Previous studies have also raised that several physical, chemical, or enzymatic-assisted treatments, such as ultrasound, microwave, acid, alkali, and cellulase, could be applied for degrading polysaccharides into low-Mw fragments, thereby, influencing their antioxidant abilities (Karaki et al., 2016; Wang J. Q. et al., 2016; Mirzadeh et al., 2020; Chen X. H. et al., 2021). Yan et al. (2016a) physically degraded a *P. linteus* mycelia-derived polysaccharide using ultrasound to garner a low-Mw and low-intrinsic-viscosity fraction, performing the stronger hydroxyl radical scavenging capacity and higher values of Trolox equivalent antioxidant capacity (TEAC) and ferric-reducing ability of plasma (FRAP). Wang Z. B. et al. (2014) extracted and partially purified three polysaccharides from the *P. linteus* mycelia using hot water, 1% (NH_4_)_2_C_2_O_4_, and 1.25 M NaOH/.05% NaBH_4_ and found that the polysaccharide with higher amounts of lower Mw fractions and uronic acid contents showed the stronger DPPH radical-scavenging capacity. However, contradictory findings also exist. Yuan et al. (2018) covered that a polysaccharide from the *P. igniarius* mycelia with high Mw and intrinsic viscosity could effectively scavenge hydroxyl radicals, partly eliminating DPPH radicals and chelate ferrous ions. Besides, El Enshasy and Hatti-Kaul (2013) pointed out that the low-Mw polysaccharides could penetrate the immune systems and demonstrate stimulatory activity, whereas the advantage of high-Mw polysaccharides might be ascribed to the better binding affinity of the receptors of immune cells.

### Monosaccharide composition

Polysaccharides can be divided into homo- and hetero-polysaccharides duo to the various monosaccharides in compositions. For heteropolysaccharides consisting of a series of monosaccharides mainly embracing Glc, GlcA, Gal, GalA, Ara, Fuc, Fru, Man, Rha, and Xyl, their structures and chemical properties could be affected by the different types and sequences of monosaccharides and glycosidic bonds (Lo et al., 2011; Ren et al., 2019). Confirmation of monosaccharide composition normally involves the liberation of monosaccharides through hydrolysis and derivatization (Zhang et al., 2020; Liu et al., 2021), then subsequent measurement of the liberated monosaccharides *via* various detection techniques, including capillary electrophoresis (Baker et al., 2008), TLC (Liu and Wang, 2007), HPLC (Kim G. Y. et al., 2006; Wan et al., 2020), GC (Luo et al., 2010; Pei et al., 2015), GC-MS (Yang et al., 2007; Suabjakyong et al., 2015), HPAEC (Yang et al., 2009; Wu et al., 2013; Jin et al., 2016), etc. A neutral polysaccharide was isolated and purified from the cultured mycelia of *S. sanghuang* by DEAE Sepharose Fast Flow and Sephacryl S-100 columns (Cheng et al., 2020b). This polysaccharide was primarily composed of Glc, indicated *via* acid hydrolysis and GC-MS detection, as well as having the potential inhibitory activities against α-amylase and α-glucosidase and hypoglycemic effects on *in vitro* insulin resistance of HepG2. Jin et al. (2016) employed hot water extraction, delignification, ethanol precipitation, DEAE-52 cellulose anion-exchange, and Sephadex G-100 columns to fractionate a polysaccharide from the fruiting bodies of *P. baumii*. HPAEC analysis delineated that this polysaccharide comprised Fuc, Ara, Gal, Glc, Xyl, and Man at molar ratios of 2.19:1.27:5.85:43.22:2.73:4.18, and that Glc was the predominant monosaccharide.

Correlational studies notarized that the biological proficiency of polysaccharides is dependent on the ratios of different monosaccharides in compositions (Lo et al., 2011; Yang et al., 2020). Two proteoglycans isolated from the mycelia and fermentation broth of *P. nigricans* through submerged fermentation were elucidated to possess the similar average Mws and consist of Glc, Gal, Man, Ara, and Fuc in the molar ratios of 3.26:8.77:6.44:1:1.35 and 20.06:8.72:6.94:1:0.76, respectively. The anti-tumor and immunomodulating activities of mycelia polysaccharides were higher than those of the broth fraction, which may be related to, in part, the variations in ratios of monosaccharides in compositions (Li et al., 2008). Jia et al. (2017) separated two water-soluble polysaccharides with the similar Mws but different monosaccharide compositions from the fruiting bodies of *P. vaninii*, and by means of MTT assay identified that the alkali-extracted polysaccharide exhibited the higher inhibition effect on HepG2 and HeLa than the hot water-extracted portion *in vitro*. On reason for the results happened maybe the significant shift on monosaccharide relative contents in polysaccharides caused by the alkaline solution treatment. Similar evidence was also figured out by Jiang et al. (2015), who isolated and purified two polysaccharides from the mycelia and culture medium of *P. pini*, both neutral heteropolysaccharides comprising Man, Gal, and Glc with molecular ratios of 2.99:1.00:0.34 and 38.40:1.00:1.76, respectively. The antioxidant activities of scavenging DPPH and hydroxyl radicals, chelating ferrous ions, and reducing ferric ions of mycelia polysaccharide were stronger than those of the culture medium portion, suggesting that the antioxidant activities could be impacted by the multiple ratios of monosaccharides in compositions. Apart from bioactivities, the multiplicity in monosaccharide compositions and molar ratios may correspond to the original materials, sources, extraction, isolation, and purification methods, etc., which is authenticated by the up-mentioned examples as well.

### Chemical structure

At present, different tools, such as FT-IR, NMR spectroscopy, and the combination of methylation, periodate oxidation, Smith degradation, HPLC, GC, and GC-MS, have been devoted to clarifying the chemical structures of sanghuang polysaccharides, including α/β configurations, branching degrees, types and positions of branches and functional groups, patterns and chirality of glycosidic bonds, positions and linkage sequences of the residues in glycosidic bonds, etc. (Liu and Wang, 2007; Mei et al., 2015; Ma et al., 2019; Cheng et al., 2020a; Sun et al., 2021). FT-IR could preliminarily analyze the vibrations of atoms or functional groups existed in polysaccharides, owing to the unique wavenumber range to the adsorption bond, hereby, providing the information of anomeric configurations and glycosidic bonds (Qian et al., 2009; Ren et al., 2019). For instance, the characteristic peak at 855–833 cm^−1^ is indicative of the α configuration of Glc, whereas the higher wavenumber ranging from 905 to 876 cm^−1^ signifies the β configuration (Zhang, 1994). NMR spectroscopy has been claimed as a vital technology for almost full-structural identification of the underivatized polysaccharides, such as monosaccharide compositions, positions and patterns of branching and glycosidic linkages, and types of cyclic structures, replying on the resonance spectroscopy and Zeeman, splitting generated by the atomic nucleus in an external magnetic field after adsorbing electromagnetic waves with the certain frequencies transiting from one spin to another (Yao et al., 2021). NMR spectroscopy is also a nondestructive testing tool in which the samples are not destroyed, and the resulting data are accurate and reliable, but the “pure” polysaccharide is highly required for preparation (Gong et al., 2020). Interpretation of functional groups is based on the comparison of their chemical shifts and coupling constants with the spectral data documented in previous literature containing the relevant polysaccharides (Villares et al., 2012; Yao et al., 2021). Methylation of polysaccharides plays an important role in sample preparation in the determination of the monosaccharide compositions, anomeric configurations, occurrence of branches, and positions of glycosidic linkages (Villares et al., 2012). This overall course involves several steps: the complex carbohydrates are methylated, then hydrolyzed by acids, and the newly constructed hydroxyl groups are acetylated, then deciphered *via* HPLC, GC, or GC-MS. It is particularly noted that FT-IR is often used to examine whether the characteristic adsorption band at 3,000–3,500 cm^−1^ is completely disappeared to ensure that all the hydroxyl groups in polysaccharides are absolutely methylated (Sims et al., 2018). Periodate oxidation as a routine choice also has been used for elucidating the structures of polysaccharides referring to the cleavage of C–C bonds *via* oxidation reaction caused by periodate ion attack, accordingly, forming dialdehyde, aldehyde, or formic acid. Since each C–C bond cleavage consumes one molecule of periodate ion, the position and type of glycosidic linkages, branching degree, and chain composition, as well as aggregation, can be defined by the consumption of periodate ion and the amounts of corresponding products released (Jiang et al., 2016; Nypelö et al., 2021). Smith degradation is accomplished by the reduction of periodate-oxidized products, acid hydrolyzed, and subsequent HPLC, GC, or GC-MS analysis, whereby, inferring the linkage sequences of the monosaccharide residues in glycosidic bonds (Wasser, 2002).

Liu and Wang (2007) prepared a water-soluble polysaccharide from the fruiting bodies of *P. ribis* by hot water extraction, ethanol precipitation, DEAE-cellulose, and Superdex 30-column chromatography, and with the help of FT-IR, NMR spectroscopy, GLC-MS, methylation analysis, periodate oxidation, and Smith degradation investigated it to be a β-_D_-glucan containing a (1 → 4), (1 → 6)-joined backbone, with a single β-_D_-Glc at the *C*-3 position of (1 → 6)-joined glucosyl residue every eight residues, along the main chain. Preliminary activity tests *in vitro* revealed that this polysaccharide could stimulate the proliferation of spleen lymphocytes. A heteropolysaccharide, fractionated by Yang et al. (2009) from the fruiting bodies of *P. igniarius via* hot aqueous extraction, ethanol precipitation, DEAE-Sepharose anion-exchange, and gel filtration chromatography, was elaborated to have a skeleton consisting of 1,6-disubstituted-3-*O*-methyl-α-_D_-Glc*p* residue, 1,3,6-trisubstituted-α-_D_-Man*p* residue, 1,4-disubstituted-α-_D_-Gal*p* residue, and 1,2-disubstituted-α-_D_-Gal*p* residue, and have a 1-substituted-α-_L_-Fuc*p* terminal attached to *O*-3 of a Man*p* residue, on the basis of methylation and NMR studies (^1^H, ^13^C, COSY, TOCSY, ROESY, HSQC, and HMBC). Bioactivity assays conducted *in vitro* displayed that this heteropolysaccharide stimulated the proliferation of mice spleen lymphocytes. Jiang et al. (2016) gained a *P. pini* exopolysaccharide that contained a backbone of (1 → 2)-linked Man, being heavily substituted *via* (1 → 6)-glycosidic bonds with (1 → 3)-linked Man, and terminated mainly with Man, as well as a small amount of Gal and Glc, as affirmed by partial hydrolysis with acid, periodate oxidation and Smith degradation, methylation, and NMR spectroscopy. Immunological experiments showed that the *P. pini* exopolysaccharide demonstrated the high macrophage-activating ability, consequently augmenting phagocytosis and enhancing the production of NO, TNF-α, and reactive oxygen species (ROS). Yan et al. (2016c) using hot water extraction, ethanol precipitation, DEAE-Sepharose FF, and Sephacryl S-400 HR column chromatography purified a polysaccharide from an ammonium oxalate extract of the *P. linteus* mycelia. Results of methylation analysis, FT-IR, and NMR spectroscopy indicated that the backbone of this polysaccharide was composed of (1 → 4)-α-_D_-Glc*p*, (1 → 2)-α-_D_-Xyl*p*, and (1 → 3)-α-_D_-Ara*f* residues, whereas the (1 → 6)-α-_D_-Man*p* residues formed branches at the *O*-2 position with 1-linked-α-_D_-Glc*p* terminal residues. From the antioxidative activity measurements *in vivo*, this polysaccharide enhanced the activities of superoxide dismutase (SOD), catalase (CAT), and glutathione peroxidase (GSH-Px), and reduced the malondialdehyde (MDA) levels in serum and liver of the _D_-Gal-treated aging mice in a concentration-dependent manner, as well as effectively stimulated the immune systems of aging mice.

These findings also provide the considerably valuable clues and new insights for elaborating the structure-bioactivity relationships of sanghuang polysaccharides, that is, the complexity of structures of polysaccharides is significantly associated with their biological functionalities (Ferreira et al., 2015; Qu et al., 2020; Song et al., 2021). For instance, Ara 1 → 4 and Man 1 → 2 linkages of the branched chains in polysaccharides are positively correlated with their FRAP, whereas Glc 1 → 6 and Ara 1 → 4 linkages are related with their abilities on scavenging DPPH radicals (Lo et al., 2011). Polysaccharides abundant in the → 3)-β-_D_-Glc*p*-(1 → linkage strongly abate their inflammatory responses, and so do the polysaccharide plentiful in rich sequences of the side-chain units (Nie et al., 2017; Qu et al., 2020). An exopolysaccharide from the fermentation broth of *S. sanghuang* was purified by ethanol precipitation, DEAE-52 cellulose, and Sepharose CL-6B columns, as well as was disclosed to comprise a skeleton of → 4)-β-Man*p*-(1 → 4)-α-Ara*f*-(1 → 3,4)-α-Glc*p*-(1 → 3,4)-α-Glc*p*-(1 → 3,4)-α-Glc*p*-(1 → 3,4)-α-Glc*p*-(1 → 3,4)-α-Glc*p*-(1 → 6)-α-Gal*p*-(1 → 4)-β-Man*p*-(1 → and five branches, including four α-_D_-Glc*p*-(1 → and one α-_D_-Man*p*-(1 → with the aid of FT-IR, methylation, and ^1^H, ^13^C, COSY, DEPT, TOCSY, ROESY, HSQC, and HMBC NMR spectroscopy, and, hence, increase antioxidant enzymes activities and diminish lipofuscin levels, and ameliorate histopathological hepatic lesions and apoptosis in hepatocytes of the _D_-Gal-aged mice (Ma et al., 2019). Sun et al. (2021) isolated a 46-kDa heteropolysaccharide from the fruiting bodies of *P. baumii via* hot water extraction, ethanol precipitation, DEAE-Sepharose Fast Flow, and Sephadex G-200 gel-permeation column chromatography, and found it had a backbone containing 1,3-linked β-_D_-Glc*p* and 1,6-linked α-_D_-Gal*p* residues, being attached to Araf, Man*p*, and Gal*p* units as oligosaccharidic side chains to the skeleton at *C*-6 position of some Glc*p*. Moreover, this polysaccharide displayed markedly anti-inflammatory activity in the LPS-stimulated macrophage RAW264.7 cells by downregulating phosphorylation of the signal transducer and activator of transcription 1 and increasing the tissue repairing genes, as well as alleviated the dextran sodium sulfate-inducible ulcerative colitis in mice, presumably attributed to the copious branched chains and → 3)-β-_D_-Glc*p*-(1 → linkages.

### Conformational feature

Conformation of polysaccharides is delimited as their 3D structures in liquid or solid-state through bonding or spacing physical forces resulting from their chemical structures, including spherical, random coil, single-helix, double-helix, triplex-helix, rod-like, etc. (Zhang et al., 2020; Guo X. Y. et al., 2021). Likewise, biological activities of polysaccharides are drastically affected by their conformational features. More flexible and extended conformation, as well as good solubilities, confer polysaccharides to have more opportunities to bind to cell membranes and have stronger interactions with the correlated receptors, which lead to the higher antioxidant, anti-tumor, and immunomodulatory activities (Falch et al., 2000; Zheng et al., 2017; Guo X. Y. et al., 2021). Based on the data of Mw and monosaccharide composition, Wang Z. B. et al. (2014), making use of DLS and Congo Red test, proved the three partially purified polysaccharides extracted from the *P. linteus* mycelia using hot water, acid, and alkali to exist as compact random coils in aqueous solutions. *In vitro* antioxidant assays verified that the acid- and alkali-extracted polysaccharides exerted the stronger scavenging capacities and antioxidant activities in a dose-dependent manner, imputed to the chemical structures and conformational features. Jia et al. (2017) illustrated that one fraction isolated from the fruiting bodies of *P. vaninii* by hot water was a high-branched heteropolysaccharide with a globular shape, whereas another fraction extracted by NaOH solution was a β-1,3-_D_-glucan branched with β-1,6-_D_-Glc and adopted more extended and flexible random coils conformation, as checked by FT-IR, acid hydrolysis and GC-MS, ^13^C NMR spectroscopy, and SEC-MALLS-RI and capillary viscometer. MTT assay indicated that the NaOH-treated polysaccharide had the stronger inhibition activity against HeLa and HepG2 *in vitro*, which revealed that the presence of β-glucan, high chain rigidity, good water solubility, and moderate Mw of the derivatives in an aqueous solution were beneficial to the increase in cytotoxicity on the growth of tumors. A polysaccharide was obtained by Yuan et al. (2018) from the *P. igniarius* mycelia, and was elucidated to exhibit a triple helical structure and have a high Mw, a high intrinsic viscosity, and a linear repeating backbone composing of Glc*p*, Gal*p*, and Man*p* joined by α-(1 → 4), α-(1 → 3), and α-(1 → 6) linkages, and single α-terminal-_D_-Glc*p* as side chains of 6-*O*-connected to the main chain through HPGPC, capillary viscometer, acid hydrolysis and HPLC, FT-IR, methylation analysis and GC-MS, NMR spectroscopy, and Congo Red test. Consequences of the antioxidant and anti-tumor tests delineated that this polysaccharide with triple helix conformation, and only α-type glycosidic bond promoted the positive scavenging effects against DPPH and hydroxyl radicals, and ferrous ions chelating activity *in vitro*, as well as possessed the potent anti-tumor activity against the growth of HT29 and MCF7 *in vitro*. Cheng et al. (2020a,b) isolated two polysaccharides from the liquid culture broth and mycelia of *S. sanghuang*. In addition to the tests of acid hydrolysis and GC-MS, FT-IR, methylation analysis and GC-MS, and NMR spectroscopy (^1^H, ^13^C, COSY, TOCSY, HSQC, HMBC, and NOESY), the two polysaccharides were ascertained to exist in flexible chain conformation in 0.1 M NaNO_3_ by using AFM, SEC-MALLS-RI, and 3D molecular modeling. Further, the two polysaccharides showed the potential for either anti-tumor or hypoglycemic effects.

From what was described above, it can be seen that deeper and accurate identifying the conformational features of sanghuang polysaccharides contributes helpfully to probe their biological and functional activities, even in designing biomaterials for clinic therapy and vaccines (Zafar et al., 2016; Makvandi et al., 2020; Alturki, 2021; Prateeksha et al., 2021). There have been many powerful techniques applied in explaining the architecture of an individual polysaccharide, including the Congo Red test, SEC-MALLS-RI, capillary viscometer, AFM, XRD, molecular modeling, circular dichroism, DSC, DLS, etc. The Congo Red test is among the commonly used method because of the low demand of operation and the needlessness of advanced instrument. Congo Red, an acidic dye, can bind with the triple helical polysaccharides to form complexes, which induce a characteristic birefringence shift in the adsorption wavelength when compared with the original Congo Red, therefore, reflecting the presence of triple-helix structures of polysaccharides (Ogawa et al., 1972; Wang Z. B. et al., 2014; Guo X. Y. et al., 2021). Conformational features of the polysaccharides can also be incarnated by some latent parameters, such as intrinsic viscosity (η), hydrodynamic radius (*R*_h_), radius of gyration (*R*_g_), molar mass per contour length (*M*_L_), persistence length (*q*), chain diameter (*d*), Huggins constant (*k*′), and Mark-Houwink equation exponent (α), which can be monitored through SEC-MALLS-RI and capillary viscometer and calculated on the basis of related equations (Jia et al., 2017; Cheng et al., 2020a,b; Guo X. Y. et al., 2021). AFM has emerged as a visual tool for investigating the shape, size, and aggregated morphology of macromolecular chains to further characterize the conformation of polysaccharides (Cheng et al., 2020a,b; Guo X. Y. et al., 2021). Lately, molecular modeling plays a novel role in predicting the 3D structures and exploring the mechanisms of polysaccharides by mimicking the spatial conformation of biological macromolecules and their intramolecular interactions (Cheng et al., 2020a; Guo X. Y. et al., 2021).

### Biological activities

Nowadays, the worldwide population is rapidly aging so considerable attention had been paid to a balanced diet and health needs. People are urgent to find more alternative active ingredients in our daily food and health care products, and sanghuang polysaccharides are in line with this (Aida et al., 2009; Yang M. Y. et al., 2019; Li N. Y. et al., 2021; Martinez-Medina et al., 2021; Yadav and Negi, 2021). Innumerable works, *in vitro* and *in vivo*, have been implemented to substantiate that the sanghuang polysaccharides have multiple biological activities and health benefits, such as antioxidant and anti-aging (Wang et al., 2014a; Yuan et al., 2018; Zuo et al., 2021), anti-tumor and immunomodulatory (Chen et al., 2011; Liu et al., 2018; Wan et al., 2020), anti-inflammatory and anti-nociceptive (Li et al., 2015; Wang et al., 2019; Sun et al., 2021), anti-diabetic (Kim et al., 2010; Wu et al., 2016; Khursheed et al., 2020), anti-microbial (Lee S. M. et al., 2010; Reis et al., 2014), anti-fatigue (Zhong, 2020), hepatoprotective (Liu et al., 2019b; Zhang et al., 2019; Chen C. et al., 2020), neuroprotective (Liu Y. H. et al., 2015; Yang P. et al., 2019) activities, etc. Even though the bioactivities of sanghuang polysaccharides are intensively mined, scientific research focusing on deciphering the structure-bioactivity relationships and underlying mechanisms is still relatively insufficient. The available information regarding the biological activities of sanghuang polysaccharides are provided in Supplementary Table 2 and discussed in detail below.

### Antioxidant and anti-aging activities

It is widely accepted that ROS, including hydroxyl radical (·OH), superoxide anion (·O${}_{2}^{-}$), and hydrogen peroxide, produced during the metabolism by normal cells play an essential part in the homeostasis in organisms (Barja, 2004; Wang J. Q. et al., 2016). Nevertheless, an excess of ROS, either credited to cellular stress and/or disordered metabolism and enzymatic systems, can trigger disfunction, cause DNA damage, and ultimately be involved in the pathogenesis of a variety of human diseases, such as aging, diabetes, rheumatoid arthritis, inflammation, cardiovascular disease, atherosclerosis, cerebral ischemia, and cancer (Benzie, 2000; Lu and Finkel, 2008; Espinosa-Diez et al., 2015; Li C. P. et al., 2021; Yuan et al., 2021). Many examples demonstrated that the sanghuang polysaccharides, which are capable of balancing the overproduction of ROS and protecting the living organisms against these diseases through capturing free radicals and/or promoting antioxidant enzymes activities, have been pursued as the potential ROS scavengers and antioxidants (Azeem et al., 2018; Forman and Zhang, 2021; He et al., 2021b). As concluded in Supplementary Table 2, sanghuang polysaccharides exhibited significant antioxidant activities in comparison with the untreated groups using both *in vivo* and *in vitro* assays, reflecting in scavenging superoxide anions and hydroxyl, DPPH, and ABTS radicals, reducing power, chelating metal ions, enhancing cell viability, the activities of SOD, CAT, and GSH-Px, and levels of ascorbic acid and TEAC, decreasing cell apoptosis, MDA content, and the activities of myeloperoxidase, alanine aminotransferase (ALT), aspartate aminotransferase (AST), and lactic dehydrogenase, upregulating the mRNA expression of IL-10, NAD(P)H quinone oxidoreductase 1 (NQO1), glutamate-cysteine ligase, catalytic subunit, and glutamate-cysteine ligase, and modifier subunit, as well as downregulating the levels of TNF-α and IL-1β mRNA. On the one hand, sanghuang polysaccharides act as the hydrogen atom donators to radicals by the weak dissociation energies of O–H bonds, then, emerging with stronger scavenging capacities against free radicals in terminating radical chain reactions. In addition, the existence of two or more of the following functional groups, such as O–H, S–H, –COOH, –PO_3_H_2_, C=O, –NR_2_, –S–, and –O–, in the structures of sanghuang polysaccharides is in favor of metal chelating activities. Antioxidant enzymes also contribute to prevent cells, cell membranes, cellular DNA, lipids, and proteins from oxidative stress. They were produced by stimulation of sanghuang polysaccharides and can catalyze the breakdown of lipid hydroperoxides and hydrogen peroxides to stable forms (Wang J. Q. et al., 2016; Cheng et al., 2018).

Xu et al. (2017) achieved the maximum yield of *P. vaninii* polysaccharide when Suc was employed as a carbon source. Likewise, Suc was also the best carbon source from the perspective of antioxidant activity, owing to the high Gal content, moderate Mw, and nearly globular shape form of the polysaccharide, as confirmed by Mw estimation, monosaccharide composition analysis, and FT-IR spectroscopy. A degraded polysaccharide from the *P. linteus* mycelia was caught through hot water extraction, (NH_4_)_2_C_2_O_4_ and NaOH/NaBH_4_ treatment, ethanol precipitation, and ultrasonication. The degraded fraction with a low Mw and viscosity, by contrast, demonstrated the stronger hydroxyl radical scavenging capacity and higher TEAC and FRAP (Yan et al., 2016a). Zhang et al. (2014) took advantage of a flat-plate ultrasound technology to harvest a polysaccharide from the *P. igniarius* mycelia, and using *in vitro* assays indicated that the polysaccharide with a lower Mw and higher amounts of carbohydrate, uronic acid, hydroxyl groups, and monosaccharide Rha had stronger antioxidant activities. The findings were in accordance with Yuan et al. (2018) and Wu Y. et al. (2022) who found that the polysaccharides from the *P. igniarius* mycelia with lower Mw and higher contents of electrophilic groups facilitated the liberation of hydrogen from O–H bonds and motivated the higher antioxidant capacities. It was suggested that strain species, sources, and culture media and extraction, isolation, and purification methods could impose great effects on the antioxidant and anti-aging properties of sanghuang polysaccharides. Besides, the activities depend chiefly on the structural characteristics, including the amounts of carbohydrate, uronic acid, and sulfate and reducing end, Mws, viscosities, monosaccharide constituents, conjugates, positions and types of glycosidic linkages and branches, etc. For example, polysaccharides with more complex structures and lower Mws, involving more side-branches and residues, could alleviate the aging process to varying extents (Ma et al., 2019). The carboxyl groups substituted at *C*-5 position of the main chain could activate the chelating effects, hence, reinforcing the antioxidant capabilities of sugars (He et al., 2012). Thus, the performance of sanghuang polysaccharide is not a behavior of a single factor but a multifaceted function of two or several factors (Benzie, 2000; Wang et al., 2013; Wang J. Q. et al., 2016; Huang et al., 2017). Furthermore, chemical modification, including sulfation, carboxymethylation, acetylation, phosphorylation, benzoylation, quaternization, and periodate oxidation, can influence the antioxidant activities of polysaccharides as well, attributed to the weaker dissociation energy of hydrogen bonds by introducing the substituted groups into their structures, and the decrease in anomeric carbon (Bedini et al., 2017; Shah et al., 2018; de Almeida and da Silva, 2021).

### Anti-tumor and immunomodulatory activities

Until now, rapid increases in morbidity of malignant tumors with high mortality have become the great challenges for global public health, characterized by the uncontrollable division and proliferation of abnormal cells (Bode and Dong, 2009; Klein et al., 2021). Even though conspicuous progress has been achieved on the treatment of carcinomas by traditional methods, including surgical interventions, radiotherapy, chemotherapy, bone marrow transplant, immunotherapy, and hormone therapy, chemotherapeutical agents endure severe side effects, such as targeting non-specificity and heavily opportunistic resistance to drugs (Schirrmacher, 2019). Hence, the quest of studying and developing new potential anti-tumor candidates, particularly derived from natural resources, is an eager demand, which not only diagnose and cure the human malignancies and directly induce the tumor cells apoptosis, but can also be functionalized as the immunomodulators to suffer from adverse stress and strengthen body's immune defense against the development of cancer cells (Moradali et al., 2007; de Silva et al., 2012; Kothari et al., 2018; Sindhu et al., 2021). Mushroom polysaccharides were first reported for anti-tumor properties by Byerrum et al. (1957). Ikekawa et al. (1968) declared that *P. linteus* showed an inhibitory rate of 96.7% against S180. Ever since, numerous sanghuang polysaccharides have been prepared, and *in vivo* animal and *in vitro* research have shown them to display striking anti-tumor and immunostimulant activities across a wide range of cancer cell lines, such as S180 (Chen et al., 2011; Mei et al., 2015), B16 (Kim G. Y. et al., 2006), H22 (Gao et al., 2017), HepG2 (Cheng et al., 2020a), Bel7402 (Pei et al., 2015), A549 (Ying et al., 2017), SGC7901 (Liu et al., 2016), HT29 (Yuan et al., 2018), SW480 (Li S. C. et al., 2015), HCT8 (Pei et al., 2015), MCF7 (Wan et al., 2020), Hela (Ying et al., 2019), etc.

A 250-kDa *P. linteus* polysaccharide, consisted primarily of repeating α-_D_-Glc(1 → 4)-α-_D_-Glc(1 → 6) units, was validated to harbor stronger anti-proliferative effect against S180 through lowering the expression level of anti-apoptotic Bcl-2 protein and increasing the level of pro-apoptotic protein Bax, which, in turn, facilitated the release of cytochrome *c* from mitochondrial intermembrane compartment into cytosol, thus, causing apoptotic cell death (Mei et al., 2015). Similar results were obtained by Li et al. (2004) and Wang G. B. et al. (2012), which also implied that the sanghuang polysaccharide-induced apoptosis was interrelated with a decrease in Bcl-2 level and an increase in the release of cytochrome *c*. Zhong et al. (2013) courted an insight into the action mechanism of *P. linteus* polysaccharide triggered-HT29 apoptosis, namely, remarkably downregulating the expression of cyclin D1, cyclin E, and CDK2, as well as increasing the expression of p27Kip, thus leading to the *S*-phase cell cycle arrest in HT29. Wan et al. (2020) extracted and characterized a water-soluble *S. vaninii* polysaccharide, which was qualified for effectively inhibiting the proliferation of MCF7, stopping the cell cycle at *G*_2_ phase, accelerating apoptosis, and reducing the migratory and invasive powers, through driving the activation of p53-related genes and downregulating the expression of matrix metalloproteinase (MMP). A sulfated polysaccharide constructed by sulfation of a glucan from the fruiting bodies of *P. ribi*, using chlorosulfonic acid protocol, exerted anti-cancer activity, which is the anti-angiogenic activity by intervention of proliferation, migration, and formation of vascular endothelial growth factor (VEGF) in EA.hy926, with the mechanism as downregulating the protein expression of VEGF and VEGF receptor-1 and restricting the phosphorylation of VEGF receptor-2, protein kinase B, and extracellular signal-regulated kinase (Liu et al., 2018). Xue et al. (2011) obtained a *P. baumii* polysaccharide and exposed it to the RAW264.7 macrophages, accompanied by noticeable increases in the cellular proliferative rate, NO production, and expression levels of IL-1β, IL-18, IL-6, IL-12p35, and IL-12p40 genes. MTT assay and cell cycle analysis suggested that this polysaccharide could markedly suppress the proliferation of HepG2 in a dosage-dependent manner by inducing the cell cycle arrest at *S* phase, incurring apoptosis. As displayed by the study of Liu et al. (2016), a homogeneous polysaccharide purified from the fruiting bodies of *P. baumii* had an antiproliferative effect on Hela and SGC7901 by mediating the block of cell division in *G*_0_/*G*_1_ and *S* phases, improvement of immune cell phagocytic activity, and secretion of TNF-α and IL-6. Two polysaccharides, isolated by Li et al. (2008) from the *P. nigricans* mycelia through submerged fermentation and culture medium, both owned the anti-tumor activties against S180 *in vivo*. Further experiments certified that no direct cytotoxic activity against S180 was monitored, but apparent increases in lymphocytes proliferation and production of NO and TNF-α in microphages were observed, indicating that anti-cancer effects of the two carbohydrates are not directly tumoricidal but rather immunostimulating. Tremendous evidence have authenticated that the possible anti-tumor mechanisms of sanghuang polysaccharides are involved in the direct cancer inhibition activities, including cell-cycle arrest, induction of apoptosis, anti-angiogenesis, and inhibition of metastasis, including proliferation, invasion, migration, and adhesion (Zhang et al., 2007; Khan et al., 2019; Hyder and Dutta, 2021). More importantly, sanghuang polysaccharides can assist a host to boost the immunomodulatory activities to fight against cancer cells, so-called as biological response modifiers. They rely on either the direct activation of various immune cells like macrophages, T-lymphocytes, B-lymphocytes, natural killer cells, and dendritic cells, or potentiation of the release of various cell signal messengers, inflammatory mediators, and cytokines, such as IL-2, IL-4, IL-10, IL-1β, IFN-γ, IFN-β, TNF-α, cyclooxygenase-2, and NO (Wasser, 2002; Zhu et al., 2007; Lin et al., 2016; Singdevsachan et al., 2016; Chakraborty et al., 2019; Yin et al., 2021).

It is well-documented that the anti-cancer activities of sanghuang polysaccharides are notably decided by their refined structures in all hierarchies, such as Mws, chemical compositions, configurations of glycosidic linkages, backbones and branched chains, types of substituent groups, 3D conformation, etc. (Zhang et al., 2007; Meng et al., 2016; Yan et al., 2017). Only by scrutinizing their structures may explain their properties and offer the valuable direction for purpose screening, modification, and synthesis. In general, the larger the Mws and the better water solubility of sanghuang polysaccharides, the stronger the anti-tumor activities (Li S. C. et al., 2015; Pei et al., 2015). Kim et al. (2003) got an acidic proteo-heteroglycan mixed both with α- and β-linkages, substituted with linear _D_-(1,3), branched _D_-(1,6) and terminal _D_-residues at *C*-6 position, and illuminated that the structural features like 3D conformation, branching ratio, and molecular complexity are quite crucial for the anti-tumor activity by the rapid proliferation of spleen cells and stimulation of humoral host defense and macrophage function. Moreover, *P. vaninii* polysaccharide, a β-glucan with good water solubility and relatively high chain stiffness, pose beneficial to promotion of the anti-tumor capacities by having more chances to bind to cell membrane and higher cytotoxicity to cancer cells (Jia et al., 2017).

### Anti-inflammatory and anti-nociceptive activities

Inflammation is a comprehensive, natural self-protective response of organism mediated by the actions of cells in innate immune system, to address the injury or damage caused by mechanical, chemical, or microbial stimuli, such as infections, irritants, ultraviolet light irradiation, pathogens, and allergens. Among these cells, macrophages play a pivotal role during inflammation, including the surplus of inflammatory mediators, like NO by inducible nitric oxide synthase, prostaglandin E2 by cyclooxygenase-2, and ROS, as well as the increase in expression of some pro-inflammatory cytokines, such as TNF-α, IFN-γ, IL-6, and IL-1β, which are extremely linked to a wide variety of diseases (Shi, 2016; Nie et al., 2017; Du et al., 2018; Yang P. et al., 2019; Hou et al., 2020). A large body of studies have discovered that sanghuang polysaccharides own the desired anti-inflammatory and anti-nociceptive activities. A selenium-enriched polysaccharide from the *P. igniarius* mycelia could act a positive influence on skin repairing in mice through lowering the expression of IL-6, TNF-α, and VEGF over cascade (Luo L. J. et al., 2021). Ma et al. (2019) disclosed that a polysaccharide from the *S. sanghuang* broth reinforced the immune organ efficacy and mitigated the histopathological hepatic lesions and apoptosis in hepatocytes of the _D_-Gal-aged mice in a dose-dependent behavior. Sanghuang-derived polysaccharides are capable of attenuating inflammation and pushing tissue healing by downregulating the phosphorylation level of STAT-1 and the expression level of STAT-1 targeted genes, such as inducible nitric oxide synthase, TNF-α, IL-1β, IL-2, IL-6, and IL-12 in an LPS-induced macrophage RAW264.7 model (Xie et al., 2019; Sun et al., 2021; Zuo et al., 2021). Their anti-inflammatory and anti-nociceptive effects originate from blocking and inhibiting the activation of MAPK and peroxisome proliferator-activated receptor (PPAR) signaling pathways (Hu et al., 2018; Yin et al., 2021).

### Anti-diabetic activity

Unlike other types of sugars, polysaccharides extracted from the mushrooms sanghuang do not raise the concentration of glucose in blood but possess the robust anti-diabetic activities, primarily by inhibiting the β-cells apoptosis in pancreatic islets, affecting the activities of glucose-metabolizing enzymes, such as α-amylase and α-glucosidase, and enhancing the glucose disposal, synthesis of hepatic glycogen, and insulin sensitivity, as well as activating the expression of adenosine monophosphate-activated protein kinase and PPAR-γ modulators (Wu et al., 2016; Azeem et al., 2018; Ajith and Janardhanan, 2021). One study confirmed that the treatment with a polysaccharide from the *P. linteus* mycelia could suppress the development of autoimmune diabetes in NOD mice not only through delaying the transfer of diabetes by spleen cells from NOD mice into NOD/SCID mice but also through adjusting the expression of pro-inflammatory cytokines, including IFN-γ, IL-2, IL-4, and TNF-α by Th1 and Th2 cells (Kim et al., 2010). Oral administration with a water-soluble crude polysaccharide from the *P. linteus* mycelia at 100 mg/kg body weight/d could distinctively cut down the blood glucose level by 35.60% in the alloxan-revulsive diabetic mice (Zhao et al., 2014). Another report warranted that a neutral polysaccharide from the *S. sanghuang* mycelia owned the potential inhibitory activities against α-amylase and α-glucosidase and had the hypoglycemic impacts on *in vitro* insulin resistance of HepG2, implying that this polysaccharide may be suitable as the functional anti-diabetic drugs for therapeutic cure (Cheng et al., 2020b). In-depth study suggested that many various molecules, such as protein kinase B, insulin receptor substrate-2, and fork head transcription factor-1, have engaged in regulating the intracellular procedure of insulin signals inside cells (Wang P. C. et al., 2016; Wu et al., 2016; Feng et al., 2018).

### Hepatoprotective activity

Abundant investigations have proven that sanghuang polysaccharides can be the hepatoprotective agents by virtue of enhancing liver metabolism, reducing the levels of lipid peroxidation and expression of inflammatory mediators, restraining oxidative stress and histopathological fibrogenesis, inducing apoptosis and cycle arrest in hepatic stellate cells, as well as improving the activities of antioxidative enzymes (Shan et al., 2019; Yuan et al., 2019; Qu et al., 2020). Liu et al. (2019b) verified that the extracellular *I. hispidus* exopolysaccharide from fermentation broth performed the liver protective ability on acute alcoholic liver injury in mice with the help of extending the duration of righting reflex, shortening the duration of recovery, and decreasing the liver index and levels of ALT, AST, and MDA, as well as increasing the levels of alcohol dehydrogenase, CAT, and SOD. Deeper research reported that this polysaccharide was able to activate the nuclear factor E2-related factor 2 signaling pathway, and increase the mRNA expression levels of downstream-related antioxidant enzymes, such as CAT, Cu-Zn SOD, and NQO1. Compared with the untreated groups, the *P. linteus* polysaccharide treatment harbored a notable reduction in liver fibrosis in a thioacetamide-induced liver damage mice model *via* regulation of the oxidative stress pathways, heat shock pathways, and metabolic pathways, referring to a total of 13 differentially expressed proteins, including actin, tubulin α-1C chain, preprohaptoglobin, hemopexin, galectin-5, glutathione S-transferase α4, branched chain keto acid dehydrogenase heterotetrameric E1 subunit-α, glutathione S-transferase κ, glyceraldehyde-3-phosphate dehydrogenase, thiosulfate sulfurtransferase, betaine-homocysteine S-methyltransferase 1, quinoid dihydropteridine reductase, and ribonuclease UK114 (Wang H. L. et al., 2012). Chen C. et al. (2020) evaluated the hepatoprotective effect of an isolated *P. linteus* polysaccharide against the acetaminophen-stimulated liver injury in mice, which was attributed to its antioxidant functions, by decreasing cytochrome P450 2E1 expression and hepatic release of cytokines, improving the levels of phase II enzymes, exhibiting hepatic-repairing performance, and accelerating metabolism of acetaminophen.

### Anti-microbial activity

Sanghuang polysaccharides are known to show the potential of anti-bacterial, anti-fungal, and anti-viral activities, whose assessment is a valuable concept for the rational design to targeted drugs for infectious diseases (Bach et al., 2019; Liu Z. H. et al., 2020; Guo Y. X. et al., 2021; Wang Z. C. et al., 2021). Reis et al. (2014) tested the anti-microbial effects of a polysaccharide extracted from *P. linteus* against an array of Gram-positive and Gram-negative bacteria and filamentous fungi. It was observed that the *P. linteus* polysaccharide powerfully inhibited the proliferation of *Staphylococcus aureus, Bacillus cereus, Micrococcus flavus, Listeria monocytogenes, Pseudomonas aeruginosa, Salmonella typhimurium, Escherichia coli, Enterobacter cloacae, Aspergillus fumigatus, A. versicolor, A. ochraceus, A. niger, Trichoderma viride, Penicillium funiculosum, Pe. ochrochloron*, and *Pe. verrucosum*. Two polysaccharides purified from *P. pini* exerted the anti-viral activities against coxsackie virus B3, a non-enveloped virus, and herpes simplex virus-1, an enveloped virus, as well as possibly against influenza viruses, together with the decreases in neuraminidase activities in a concentration-dependent manner, revealing that these polysaccharides can be applied for antivirals after herpesvirus infection (Lee S. M. et al., 2010). More noteworthy, the abilities of polysaccharides derived from natural resources, especially mushrooms sanghuang, to modulate the immune response and cyto-protect pulmonary, make them the latent candidates for combination therapy in severe cases of COVID-19 (Chen R. R. et al., 2020; Chen X. Y. et al., 2020; Barbosa and de Carvalho Junior, 2021).

### Other bioactivities

Except for the biological activities mentioned above, sanghuang polysaccharides have also possessed other efficacies, principally including anti-fatigue activity, neuroprotective activity, prebiotic activity, etc., which are as followed. Zhong (2020) affirmed that a polysaccharide from the fermentation broth of *P. igniarius* had the conspicuous anti-fatigue impacts on sports fatigue. Liu Y. H. et al. (2015) and Yang P. et al. (2019) both found the neurotrophic activities of the polysaccharides isolated from the fruiting bodies of *P. ribis* through facilitating the neurite outgrowth of nerve growth of the factor-stimulated PC12 and inhibiting cell apoptosis, meaning they are the promising choices for treatment of neurodegenerative diseases, such as Alzheimer's. The mechanism was associated with the attenuation of PC12 death by increasing the MMP level and decreases in the protein expression of cytochrome *c*. Many investigations have validated that intake of sanghuang polysaccharides with lower digestibility in gastric acidity could be helpful for the use of probiotics, increases in the number of probiotics, amelioration of gut microbiota, and treatment of gastrointestinal diseases (Liu L. Q. et al., 2019; Niu et al., 2021; Song et al., 2021).

Additionally, sanghuang polysaccharides have received more and more attention from the therapeutic effects against cerebral ischemia, where the mechanism includes resisting oxidative stress, relieving inflammation and deactivation of the related signaling pathways and pro-inflammatory cytokines, inhibiting neurotoxicity, provoking the expression of anti-apoptotic proteins, holding mitochondrial homeostasis, and motivating cerebral angiogenesis (Meng et al., 2021; Yuan et al., 2021).

Notably, the versatile functions of sanghuang polysaccharides primarily reply on their antioxidant activities. The reason is that by upregulating the antioxidant capacities of quenching free radicals and/or accelerating antioxidant enzyme activities, sanghuang polysaccharides can withstand the oxidative stress, aging, carcinoma, inflammation, diabetes, bacteria, virus, fatigue, liver damage and neurodegenerative, gastrointestinal, cardiovascular, and cerebral diseases, as well as enhance the immunostimulating abilities (Benzie, 2000; Wang J. Q. et al., 2016; Huang et al., 2017).

## Conclusion and future perspectives

Sanghuang, distinguished as one of the most popular medicinal mushrooms in traditional Chinese medicine, has fascinated humans for centuries since it can resist and remedy dysentery, metrorrhagia, amenorrhea, aging, poisoning, digestion, allergy, and cancer. Polysaccharides are among the major ingredients of sanghuang responsible for multiple bioactivities, including antioxidant and anti-aging, anti-tumor and immunomodulatory, anti-inflammatory and anti-nociceptive, anti-diabetic, anti-microbial, anti-fatigue, hepatoprotective, and neuroprotective activities, which have earned huge interest and attention as a potential source of various therapeutic drugs, vaccines, and functional foods.

In this historical and scientific background, this review provides a systematic and thorough description of the evolution of species and their names, preparation, structural characterization, and related bioactivities of sanghuang polysaccharides, and builds upon a valuable and profound guidance for the development and utilization of natural resources-based nutraceuticals, pharmaceuticals, and cosmeceuticals. An ever-increasing number of bioactive polysaccharides have been isolated from the fruiting bodies, cultured mycelia, and fermentation broth of sanghuang species. The structural features of these carbohydrates, including Mws, sugar compositions, configurations and patterns of isomers, backbone structures, types and numbers of glycosidic linkages, positions and degrees of branching, as well as 3D conformation, have been deciphered. Although the significant progress has been garnered focusing on the sanghuang polysaccharides, there remain some issues required to be desperately addressed.

First, the taxonomy on synonym and homonym of the species names in sanghuang often confuses their utilization, thus, anxiously desiderating unification and normalization of these mushrooms. As well-known complex macromolecules, polysaccharides from different sources of raw strains, and preparation strategies can result in the differentiation of chemical properties, structural characteristics, and bioactivities due to the physical, chemical, and/or biochemical mechanisms, so hunting for new sanghuang resources and artificial cultivation contributes to their healthy utilization. Current extraction, isolation, and purification protocols largely confine to the analytical or preparative purposes in laboratory scales, and they may not be reasonable for each kind of polysaccharide. New, feasible, economic, and efficient strategies should be developed, and a related database for the optimized cultivation and preparation of polysaccharides derived from each sanghuang species should be established for standardization, industrialization, and commercialization. More attempts regarding the genetic and metabolic engineering should be conducted to harvest the higher yield of sanghuang polysaccharides, lower cost, shorten cycle, and reuse wastes.

The bioactivities of sanghuang polysaccharides are the results of a combination of various factors. Although many chemical structures and bioactivities of sanghuang polysaccharides and their derivatives have been elucidated, understanding of the relationships between structures and bioactivities, as well as the accurate impactful factors and cellular and molecular mechanisms of actions underlying their bioactivities still lack depth and precision. It is of imperative importance to construct the analytically high-resolution techniques and deploy the massive *in vitro* and *in vivo* experiments, as well as clinical trials, to further guarantee the consistency and reliability, which is vital to control the usage quality and structural characteristics of sanghuang polysaccharides. Another meaningful route is by means of modification, including physical (ultrasonication, microwave treatment, radiation exposure, steam explosion, hyperthermy, high pressure homogenization, and plasma copolymerization), chemical (sulfation, phosphorylation, carboxymethylation, selenization, methylation, acetylation, benzoylation, quaternization, and periodate oxidation), or biological (enzymatic degradation) treatment to clarify the structure-activity relationships; a steerable polysaccharide will be created and its properties can be predicted (Karaki et al., 2016; Xu et al., 2019).

Previous studies have validated that the administration of sanghuang extracts seems to be non-toxic or hypotoxic (Huo et al., 2020; Li I. C. et al., 2020). For efficient uses in nutraceuticals, pharmaceuticals, and cosmeceuticals, the assessment of safety, adverse effects, and efficacies of sanghuang polysaccharides is imminently necessitated in deciding the normative dosage and administration duration in the next steps, such as acute oral toxicity test, chromosome aberration test, bone marrow micronucleus test, and clinical test.

Discovery of other novel activities of sanghuang polysaccharides is a fresh frontier, which can be combined with other materials to improve their characteristics and be reflected in other physical properties, entitling them as the safe and effective additives. The combination of artificial materials, such as nanocomposites, can perfectly enhance the non-toxicity, surface area, tensile strength, biocompatibility, biodegradability, and environmental adaptation, imparting them to exhibit the ideal features, and be subjected to the packing biomaterials, emulsions, and immunostimulants for carrying and maintaining the functionalities of foods and drugs (Mohan et al., 2019; Zhang R. Y. et al., 2020; Chen C. W. et al., 2021; Luo M. C. et al., 2021; Mahendiran et al., 2021; Zhang et al., 2021). Also, a database with respect to the sources, structures, active sites, bioactivities, involved mechanisms, and structure-activity relationships of sanghuang polysaccharides should be set up to better design delivery systems, as well as accelerate scientific and rational protection, development, and application of the medicinal mushrooms sanghuang.

## Author contributions

B-KC and JS conceived and designed manuscript. HW, J-XM, and MZ collated references. JS and HW wrote manuscript. All authors had reviewed this manuscript before submission.

## Funding

This work was supported by the National Natural Science Foundation of China (Nos. U2003211 and 32070016), the Scientific and Technological Tackling Plan for the Key Fields of Xinjiang Production and Construction Corps (No. 2021AB004), and the Beijing Forestry University Outstanding Young Talent Cultivation Project (No. 2019JQ03016).

## Conflict of interest

The authors declare that the research was conducted in the absence of any commercial or financial relationships that could be construed as a potential conflict of interest.

## Publisher's note

All claims expressed in this article are solely those of the authors and do not necessarily represent those of their affiliated organizations, or those of the publisher, the editors and the reviewers. Any product that may be evaluated in this article, or claim that may be made by its manufacturer, is not guaranteed or endorsed by the publisher.
