# Supplementary material for: Current advances and potential trends of the polysaccharides derived from medicinal mushrooms sanghuang
Source: Front Microbiol. 2022 Aug 3;13:965934. doi: 10.3389/fmicb.2022.965934 (PMC9382022; doi:10.3389/fmicb.2022.965934)
Supplement: Supplementary file 1 [file Table_1.doc]

**SUPPLEMENTARY TABLE 1.** Summary of the polysaccharides from different sanghuang species reported in recent years and their integrated information in terms of strains, sources, extraction, isolation, and purification methods, molecular weights, monosaccharide compositions, and structural features.

| **No.** | **Compound name** | **Strain** | **Source** | **Extraction and isolation methods** | **Purification method** | **Mw** | **Monosaccharide composition** | **Structural feature** | **Reference** |
| --- | --- | --- | --- | --- | --- | --- | --- | --- | --- |
| 1 | Not given | *Phellinus igniarius* | Fermentation with soybeans | Hot water extraction and ethanol precipitation | ND | ND | ND | ND | Shon and Nam, 2002 |
| 2 | Fr-I | *Phellinus linteus* | Fermentation broth | Ethanol precipitation | Sepharose CL-4B chromatography | 4.334 × 10^5^ g/mol | Rib:Man:Gal:Glc = 14.64:41.55:25.19:18.62 | Existing as a spherical form in an aqueous solution | Hwang et al., 2003 |
| 3 | Fr-II |  |  |  |  | 3.147 × 10^4^ g/mol | Fuc:Ara:Xyl:Man:Gal:Glc = 4.03:2.54:2.98:15.87:31.15:43.43 | Existing as a random coil in an aqueous solution |  |
| 4 | Fr-III |  |  |  |  | 1.295 × 10^4^ g/mol | Rib:Man:Gal:Glc = 8.89:10.76:6.67:73.68 | ND |  |
| 5 | Not given | *Phellinus linteus* | Fruiting bodies | Hot water extraction and ethanol precipitation | DEAE-Cellulose chromatography and Sepharose CL-4B chromatography | 150 kDa | Man, Gal, Glc, Ara, and Xyl | An acidic proteo-heteroglycan mixed both *α*- and *β*-linkages, and substituted with linear _D_-(1,3), branched _D_-(1,6), and terminal _D_-residues at *C*-6 position | Kim et al., 2003 |
| 6 | Not given | *Phellinus linteus* | Fermentation broth | Ethanol precipitation | ND | ND | ND | ND | Shon and Nam, 2003 |
| 7 | Not given |  | Mycelia | Hot water extraction and ethanol precipitation | ND | ND | ND | ND |  |
| 8 | PPC | *Phellinus linteus* | Fruiting bodies | Hot water extraction and ethanol precipitation | DEAE-Cellulose chromatography and Sepharose CL-4B chromatography | 73 kDa | Glc:Man:Gal:Fru:GlcN = 61.9:19.5:5.5:4.9:2.4 | ND | Kim G. Y. et al., 2006 |
| 9 | PRP | *Phellinus ribis* | Fruiting bodies | Hot water extraction and ethanol precipitation | DEAE-Cellulose chromatography and Superdex 30 chromatography | 8.59 kDa | Glc | Existence of a (1→4), (1→6)-linked backbone, with a single *β*-_D_-Glc at *C*-3 position of (1→6)-linked glucosyl residue every eight residues | Liu and Wang, 2007 |
| 10 | PIP60-1 | *Phellinus igniarius* | Fruiting bodies | Hot water extraction and ethanol precipitation | DEAE-Sepharose Fast Flow chromatography and High-Resolution Sephacryl S-1000 gel-permeation chromatography | 17.1 kDa | Fuc:Glc:Man:Gal:3-*O*-Methyl-_D_-Gal = 1:1:1:2:1 | Backbone composed of *α*-_L_-Fuc*p*-(1→ residues, *α*-_D_-Glc*p*-(1→ residues, →2,6)-*α*-_D_-Man*p*-(1→ residues, →6)-*α*-_D_-Gal*p*-(1→ residues, →2,6)-3-*O*-Methyl-*α*-_D_-Gal*p*-(1→ residues, →2,4)-3-*O*-Methyl-*β*-_D_-Gal*p*-(1→ residues | Yang et al., 2007 |
| 11 | Not given | *Phellinus linteus* | Fruiting bodies | Hot water extraction | Sepharose CL-6B chromatography | 25 kDa | Glc, Man, Gal, and Xyl | Backbone composed of *β*-(1→3) glucomannan with a core of *β*-(1→3)-linked Glc*p* residues and side branches of *β*-(1→3)-linked Man*p* residues | Baker et al., 2008 |
| 12 | PNM1 | *Phellinus nigricans* | Fermentation broth | Ethanol precipitation | DEAE-Cellulose chromatography and Sepharose CL-6B chromatography | 33 kDa | Glc:Gal:Man:Ara:Fuc = 20.06:8.72:6.94:1:0.76 | ND | Li et al., 2008 |
| 13 | PNW1 |  | Mycelia | Hot water extraction and ethanol precipitation |  | 29 kDa | Glc:Gal:Man:Ara:Fuc = 3.26:8.77:6.44:1:1.35 |  |  |
| 14 | PBF4 | *Phellinus baumii* | Fruiting bodies | Hot water extraction and ethanol precipitation | DEAE-Sepharose Fast Flow chromatography and High-Resolution Sephacryl S-1000 gel-permeation chromatography | 1.41 × 10^3^ kDa | Fuc:Glc = 1:4 | Backbone composed of *α*-(1→4)-_D_-Glc*p* with some insertions of *α*-(1→2)-_L_-Fuc*p* residues, as well as *β*-(1→4,6)-linked _D_-Glc*p* residues and *β*-glycosidically linked nonreducing end _D_-Glc*p* residues | Ge et al., 2009a |
| 15 | PBF2 | *Phellinus baumii* | Fruiting bodies | Hot water extraction and ethanol precipitation | DEAE-Sepharose Fast Flow chromatography and High-Resolution Sephacryl S-1000 gel-permeation chromatography | 2 × 10^3^ kDa | Fuc, Man, and Glc | Backbone composed of *a*,*β*-(1→6)-_D_-Glc*p*, branched with a fucosyl unit at *O*-3 position of the 3,6-di-*O*-substituted-_D_-glucosyl units and a minor (1→3,6)-*β*-_D_-Man residue and terminal Glc residues | Ge et al., 2009b |
| 16 | EPS | *Phellinus baumii* | Fermentation broth | Ethanol precipitation | ND | ND | ND | ND | Luo et al., 2009 |
| 17 | PISP1 | *Phellinus igniarius* | Fruiting bodies | Hot water extraction and ethanol precipitation | DEAE-Sepharose Fast Flow chromatography and High-Resolution Sephacryl S-1000 gel-permeation chromatography | 22 kDa | Fuc:Glc:Man:Gal:3-*O*-Methyl-_D_-Gal = 1:1:1:2:1 | Backbone composed of 1,6-disubstituted-3-*O*-methyl-*α*-_D_-Gal*p* residue, 1,3,6-trisubstituted-*α*-_D_-Man*p* residue, 1,4-disubstituted-*α*-_D_-Gal*p* residue, and 1,2-disubstituted-*α*-_D_-Gal*p* residue, and a 1-substituted-*α*-_L_-Fuc*p* terminal attached to *O*-3 position of a Man*p* residue | Yang et al., 2009 |
| 18 | IPS | *Phellinus igniarius* | Mycelia | Hot water extraction and ethanol precipitation | ND | ND | Gal | Containing *β*-glycosidic linkages | Guo et al., 2010 |
| 19 | PLP | *Phellinus linteus* | Mycelia | Hot water extraction and ethanol precipitation | DEAE-Cellulose chromatography and gel-permeation chromatography | 153 kDa | Ara:Xyl:Glc:Gal:Man = 7.0:3.7:21.1:24.1:44.2 | ND | Kim et al., 2010 |
| 20 | EP-AV1 | *Phellinus pini* | Fruiting bodies | Hot water extraction and ethanol precipitation | Sepharose CL-4B chromatography | 1.006 × 10^5^ kDa | Fuc:Gal:Xyl:Glc:Man = 4.6:19.2:17.0:53.4:5.8 | ND | Lee S. M. et al., 2010 |
| 21 | EP-AV2 |  |  |  |  | 100 kDa | Fuc:Gal:Xyl:Glc:Man = 5.1:10.3:20.1:56.1:8.4 |  |  |
| 22 | PBMP | *Phellinus baumii* | Mycelia | Hot water extraction and ethanol precipitation | ND | ND | Glc:Gal:Fuc:Man:Rha = 12.74:1.39:1.00:1.92:0.22 | Containing _D_-pyranoses | Luo et al., 2010 |
| 23 | PIE | *Phellinus igniarius* | Mycelia | Hot water extraction and ethanol precipitation | Sepharose G-100 gel chromatography | 12 kDa | Xyl:Man:Fuc:Glc:Gal = 2.3:1:6.4:22.1:19.83 | Inexistence of glucosidic bonds of *α*(1→4) | Chen et al., 2011 |
| 24 | PB | *Phellinus baumii* | Mycelia | Hot water extraction and ethanol precipitation | DEAE-Sephadex A-50 chromatography and Sephadex G-75 gel-filtration chromatography | 17 kDa | ND | ND | Xue et al., 2011 |
| 25 | Not given | *Phellinus igniarius* | Fruiting bodies | Enzyme-catalyzed extraction, ultrasonication, hot water treatment, and ethanol precipitation | DEAE-Cellulose chromatography | ND | ND | ND | Cheng et al., 2012 |
| 26 | EPS | *Phellinus igniarius* | Fermentation broth | Ethanol precipitation | Sepharose CL-6B chromatography | 3.43 × 10^5^ g/mol | ND | Existing as a random coil in an aqueous solution | He et al., 2012 |
| 27 | PM-ESP1 | *Phellinus mori* | Fermentation broth | Ethanol precipitation | Sepharose CL-6B chromatography | 4.99 × 10^3^ g/mol | Rha:Gal:Glc:Man:GlcA = 3.5:3.3:12.5:76.3:4.6 | ND | Cao et al., 2013 |
| 28 | PM-ESP3 |  |  |  |  | 2.789 × 10^4^ g/mol | Rha:Ara:Gal:Glc:Man = 5.8:1.0:2.4:7.3:81.2 | Backbone composed of (1→4)-linked Man*p*, with branches of (1→4)-linked glucosyl residues and (1→3,4)-linked Gal*p* residues |  |
| 29 | PBF3 | *Phellinus baumii* | Fruiting bodies | Hot water extraction and ethanol precipitation | DEAE-Sepharose Fast Flow chromatography and High-Resolution Sephacryl S-1000 gel-permeation chromatography | 230 kDa | Glc | Backbone composed of (1→4)-linked *β*-_D_-Glc*p* and (1→3)-linked *β*-_D_-Glc*p* units, with a single unit *β*-_D_-Glc*p* branch substituted at *O*-6 position of the 3,6-di-*O*-substituted-_D_-glucosyl units | Ge et al., 2013 |
| 30 | Not given | *Phellinus linteus* | Fruiting bodies | Hot water extraction and ethanol precipitation | ND | ND | Glc | Backbone composed of approximately 90% *β*-(1→3)(1→6) glucan and 10% *α*-glucan | van Griensven and Verhoeven, 2013 |
| 31 | PSCPL | *Phellinus linteus* | Fruiting bodies | Hot water extraction and ethanol precipitation | ND | 22-1.7 × 10^3^ kDa | Glc:Man:Gal:GlcN = 37.4:2.2:12.6:29.5 | ND | Wu et al., 2013 |
| 32 | PL-A | *Phellinus linteus* | Mycelia | Hot water extraction, (NH_4_)_2_C_2_O_4_ treatment, and ethanol precipitation | ND | 9.75 × 10^5^ and 13.9 (60%) kDa | Glc:Man:Xyl:Ara = 8:1:1:1 | Heteropolysaccharides with random coil conformations in an aqueous solution | Wang Z. B. et al., 2014 |
| 33 | PL-N |  |  | Hot water extraction, (NH_4_)_2_C_2_O_4_ treatment, NaOH/NaBH_4_ treatment, and ethanol precipitation |  | 3.11 × 10^5^ (97%) and 15.8 kDa | Ara:Xyl:Glc:Gal = 5.5:7.8:1.8:1 |  |  |
| 34 | PL-W |  |  | Hot water extraction and ethanol precipitation |  | 3.2 × 10^5^ (63%) and 49.2 kDa | Glc:Man = 8:1 |  |  |
| 35 | Not given | *Phellinus nigricans* | Mycelia | Hot water extraction and ethanol precipitation | ND | ND | ND | ND | Wang et al., 2014a |
| 36 | PNMP 1 | *Phellinus nigricans* | Mycelia | Hot water extraction and ethanol precipitation | DEAE-Cellulose chromatography and Sepharose CL-6B chromatography | 28.4 kDa | Glc:Gal:Man:Xyl = 18.65:41.37:35.41:4.57 | ND | Wang et al., 2014b |
| 37 | PNMP 2 |  |  |  |  | 31.5 kDa | Ara:Fuc:Glc:Gal:Man:Xyl = 5.78:7.24:14.42:41.57:28.62:2.37 |  |  |
| 38 | PNMP 3 |  |  |  |  | 26.1 kDa | Ara:Fuc:Glc:Gal:Man:Xyl = 3.45:8.44:21.55:36.42:26.58:3.56 |  |  |
| 39 | PIPS | *Phellinus igniarius* | Mycelia | Ultrasonication, hot water extraction, and ethanol precipitation | ND | 2.11 × 10^4^ and 3.1 (80%) kDa | Glc:Rha:Man = 11.0:14.0:1.0 | ND | Zhang et al., 2014 |
| 40 | C-PIPS |  |  | Hot water extraction and ethanol precipitation | ND | 2.54 × 10^4^ and 3.8 (80%) kDa | Glc:Rha:Man = 9.0:3.0:1.0 | ND |  |
| 41 | PLP-I | *Phellinus linteus* | Mycelia | Hot water extraction and ethanol precipitation | DEAE-Sephadex A-50 chromatography and Sephadex G-200 chromatography | 3.1729 × 10^3^ kDa | ND | A branched-type glycan with both *α*- and *β*-linkages and a pyranoid sugar ring conformation | Zhao et al., 2014 |
| 42 | PPM | *Phellinus pini* | Mycelia | Hot water extraction and ethanol precipitation | Sepharose CL-6B chromatography | 22.0 kDa | Man:Gal:Glc = 2.99:1.00:0.34 | ND | Jiang et al., 2015 |
| 43 | PPE |  | Fermentation broth | Ethanol precipitation |  | 38.0 kDa | Man:Gal:Glc = 38.40:1.00:1.76 | Backbone composed of a (1→2)-linked Man, substituted via (1→6)-glucosidic bonds with (1→3)-linked Man, and terminated with Man and a small amount of Gal and Glc | Jiang et al., 2015, 2016 |
| 44 | IPSW-1 | *Phellinus igniarius* | Mycelia | Hot water extraction and ethanol precipitation | DEAE-52 Cellulose chromatography and Sephacryl^TM^ S-400 gel-filtration chromatography | 34.1 kDa | Glc | Containing *α*-configurations | Li S. C. et al., 2015 |
| 45 | IPSW-2 |  |  |  |  | 17.7 kDa | Glc |  |  |
| 46 | IPSW-3 |  |  |  |  | 15.1 kDa | Glc |  |  |
| 47 | IPSW-4 |  |  |  |  | 21.7 kDa | Rha:Xyl:Man:Glc:Gal = 1.29:1.21:1:43.86:1.86 |  |  |
| 48 | FP30 | *Phellinus baumii* | Fruiting bodies | Hot water extraction and ethanol precipitation | ND | 1.895 × 10^3^ and 129.3 kDa | Gal:Glc:Xyl:Man:GalA:GlcA = 27.77:278.34:4.88:29.12:6.70:11.33 | ND | Li T. T. et al., 2015 |
| 49 | FP50 |  |  |  |  | 30.89 kDa | Fuc:Gal:Glc:Xyl:Man:GlcA = 54.27:114.66:229.88:9.21:98.01:36.05 |  |  |
| 50 | FP70 |  |  |  |  | 40.33 and 19.89 kDa | Ara:Gal:Glc:Xyl:Man:GalA:GlcA = 108.33:23.65:19.22:119.71:24.38:9.46:4.32 |  |  |
| 51 | LP30 |  | Mycelia |  |  | 2.012 × 10^4^ and 1.536 × 10^4^ kDa | Ara:Gal:Glc:Xyl:Man:GalA:GlcA = 11.34:79.76:251.94:13.82:61.51:23.66:5.25 |  |  |
| 52 | LP50 |  |  |  |  | 2.106 × 10^3^ kDa | Fuc:Ara:Gal:Glc:Xyl:Man:GalA:GlcA = 16.99:14.24:63.25:391.15:33.67:126.13:9.46:11.85 |  |  |
| 53 | LP70 |  |  |  |  | 31.42, 20.16, and 19.65 kDa | Ara:Gal:Glc:Xyl:Man = 43.38:84.23:358.82:32.13:82.95 |  |  |
| 54 | SP30 |  | Solid-state fermented products |  |  | 292.6 and 20.89 kDa | Ara:Gal:Glc:Xyl:Man:GalA:GlcA = 34.13:16.61:19.37:48.94:20.29:7.02:2.33 |  |  |
| 55 | SP50 |  |  |  |  | 29.15 kDa | Rha:Ara:Gal:Glc:Xyl:Man:GalA:GlcA = 11.57:57.24:54.25:32.95:65.52:49.44:19.09:8.25 |  |  |
| 56 | SP70 |  |  |  |  | 22.75 kDa | Ara:Gal:Glc:Xyl:Man:GalA:GlcA = 107.79:25.84:25.44:123.33:26.92:11.53:3.73 |  |  |
| 57 | PRG | *Phellinus ribis* | Fruiting bodies | Hot water extraction and ethanol precipitation | DEAE-Cellulose chromatography and Superdex 30 gel chromatography | 5.16 kDa | Glc | Existence of a (1→3)-linked backbone, with a branch of two (1→6)-linked and one terminal Glc substituting at *C*-6 position every three residues | Liu et al., 2015 |
| 58 | PLPS-1 | *Phellinus linteus* | Mycelia | Hot water extraction and ethanol precipitation | DEAE-52 Cellulose chromatography and Sephadex G-100 chromatography | 250 kDa | Ara:Fuc:Gal:Glc:Xyl = 1.336:1.182:1:21.964:1 | Backbone composed of repeating *α*-_D_-Glc (1→4)-*α*-_D_-Glc (1→6) units | Mei et al., 2015 |
| 59 | PLPS-2 |  |  |  |  | 28 kDa | Ara:Fuc:Gal:Glc:Man:Xyl = 1.552:1.466:2.594:14.368:1.956:1 | Backbone composed of *α*-(1→3)-_D_-Glc and *α*-(1→6)-_D_-Glc |  |
| 60 | PL-N1 | *Phellinus linteus* | Mycelia | Hot water extraction, (NH_4_)_2_C_2_O_4_ treatment, NaOH/NaBH_4_ treatment, and ethanol precipitation | DEAE-Sephadex A-25 chromatography | 3.43 × 10^5^ kDa | Ara:Xyl:Glc:Gal = 4.0:6.7:1.3:1.0 | Backbone composed of (1→4)-linked *β*-_D_-Xyl*p* residues, (1→2)-linked *α*-_D_-Xyl*p* residues, (1→4)-linked *α*-_D_-Glc*p* residues, (1→5)-linked *β*-_D_-Ara*f* residues, and (1→4)-linked *β*-_D_-Xyl*p* residues | Pei et al., 2015 |
| 61 | Not given | *Phellinus igniarius* | Fruiting bodies | Hot water extraction and ethanol precipitation | Sep-Pak C18 Plus Light Cartridge chromatography | 18.518 kDa | Rha:Man:Ara:Gal:Xyl:Glc = 1.31:14.51:2.63:20.65:3.32:57.58 | Backbone composed of →3)-*β*-_D_-Glc*p*-(1→ and →6)-*β*-_D_-Glc*p*-(1→ and a side chain of *α*-_D_-Man*p*-(1→ | Suabjakyong et al., 2015 |
| 62 | Not given | *Phellinus linteus* |  |  |  | 20.708 kDa | Rha:Man:Ara:Gal:Xyl:Glc = 0.82:8.32:1.13:8.06:2.80:78.88 | Backbone composed of →3)-*β*-_D_-Glc*p*-(1→ and →6)-*β*-_D_-Glc*p*-(1→ |  |
| 63 | PPB-MB | *Phellinus baumii* | Fruiting bodies grown on mulberry branches | Hot water extraction and ethanol precipitation | ND | 107.4 kDa | Fuc:Man:Glc:Gal:Xyl:Ara = 11.92:29.99:21.72:26.72:5.96:4.03 | ND | Zhang et al., 2015 |
| 64 | PPB-MW |  | Fruiting bodies grown on mixed wood sawdust |  |  | 148.5 kDa | Fuc:Man:Glc:Gal:Xyl:Ara = 14.17:24.58:31.66:19.27:7.43:2.89 |  |  |
| 65 | PPB-MM |  | Fruiting bodies grown on an equal combination of the two materials |  |  | 85.9 kDa | Fuc:Man:Glc:Gal:Xyl:Ara = 9.36:34.33:22.37:21.92:5.55:6.47 |  |  |
| 66 | PPB-2 | *Phellinus baumii* | Fruiting bodies | Hot water extraction, acetic acid treatment, ethanol precipitation, and ultrasonication | DEAE-52 Cellulose chromatography and Sephadex G-100 chromatography | 28.64 kDa | Fuc:Ara:Gal:Glc:Xyl:Man = 2.19:1.27:5.85:43.22:2.73:4.18 | ND | Jin et al., 2016 |
| 67 | PPB | *Phellinus baumii* | Fruiting bodies | Hot water extraction and ethanol precipitation | DEAE chromatography, Sephacryl S400 gel chromatography, and Sephacryl S200 gel chromatography | ND | ND | ND | Liu et al., 2016 |
| 68 | SHP-1 | *Phellinus sp.* | Fermentation broth | Ethanol precipitation | Q Sepharose Fast Flow chromatography and Sephacryl S-100 chromatography | 25 kDa | ND | Backbone composed of →2,4)-*α*-_D_-Glc*p*-(1→ and →2)-*β*-_D_-Man*p*-(1→ and two terminal Glc*p* branches | Ma et al., 2016 |
| 69 | PIPs | *Phellinus igniarius* | Fruiting bodies | Enzyme-catalyzed extraction | ND | ND | ND | ND | Xu et al., 2016 |
| 70 | PL-N | *Phellinus linteus* | Mycelia | Hot water extraction, (NH_4_)_2_C_2_O_4_ treatment, NaOH/NaBH_4_ treatment, and ethanol precipitation | ND | ND | Ara:Xyl:Man:Glc:Gal = 5.7:7.7:1.1:2.3:1.0 | Containing *β*-configurations | Yan et al., 2016a |
| 71 | PL-N1 |  |  | Hot water extraction, (NH_4_)_2_C_2_O_4_ treatment, NaOH/NaBH_4_ treatment, ethanol precipitation, and ultrasonication |  |  | Ara:Xyl:Man:Glc:Gal = 6.1:7.9:1.0:2.1:1.0 |  |  |
| 72 | PL-N2 |  |  |  |  |  | Ara:Xyl:Man:Glc:Gal = 5.9:7.8:1.0:2.4:1.1 |  |  |
| 73 | PL-N3 |  |  |  |  |  | Ara:Xyl:Man:Glc:Gal = 5.9:7.1:1.0:2.7:1.2 |  |  |
| 74 | PLP1-I | *Phellinus linteus* | Mycelia | Hot water extraction and ethanol precipitation | DEAE-Sepharose Fast Flow chromatography and High-Resolution Sephacryl S-400 chromatography | 2.9 × 10^5^ kDa | Glc:Gal = 8.9:1.0 | Backbone composed of (1→4)-*α*-_D_-Glc*p* residues, with branches of (1→3)-*α*-_D_-Gal*p* residues, along with 1-linked-*α*-_D_-Glc*p* terminal residues | Yan et al., 2016b |
| 75 | PL-A11 | *Phellinus linteus* | Mycelia | Hot water extraction, (NH_4_)_2_C_2_O_4_ treatment, and ethanol precipitation | DEAE-Sepharose Fast Flow chromatography and High-Resolution Sephacryl S-400 chromatography | 13.8 kDa | Ara:Xyl:Man:Glc = 1.1:1.3:1.0:6.6 | Backbone composed of (1→4)-*α*-_D_-Glc*p*, (1→2)-*α*-_D_-Xyl*p*, and (1→3)-*α*-_D_-Ara*f* residues, with branches of (1→6)-*α*-_D_-Man*p* residues at *O*-2 position, along with 1-linked-*α*-_D_-Glc*p* terminal residues | Yan et al., 2016c |
| 76 | DMPIP | *Phellinus igniarius* | Fruiting bodies | Microwave extraction and ethanol precipitation | ND | ND | ND | ND | Gao et al., 2017 |
| 77 | HPIP |  |  |  |  |  |  |  |  |
| 78 | GPIP |  |  |  |  |  |  |  |  |
| 79 | DWPIP |  |  |  |  |  |  |  |  |
| 80 | SPIP |  |  |  |  |  |  |  |  |
| 81 | UPIP |  |  |  |  |  |  |  |  |
| 82 | PV-B | *Phellinus vaninii* | Fruiting bodies | Hot water extraction, NaOH treatment, and ethanol precipitation | ND | 5.3 × 10^5^ g/mol | Ara:Xyl:Rib:Gal:Man:Glc = 1.6:1.6:0.5:0.2:4.4:91.7 | Backbone composed of *β*-1,3-_D_-glucan branched with *β*-1,6-_D_-Glc, and existing as a more expanded flexible random coil in an aqueous solution | Jia et al., 2017 |
| 83 | PV-W |  |  | Hot water extraction and ethanol precipitation |  | 4.6 × 10^5^ g/mol | Ara:Xyl:Rib:Gal:Man:Glc = 6.4:3.2:2.9:6.3:52.2:29 | Heteropolysaccharides with *α*-Man, *β*-_D_-Man, and *β*-_D_-Glc, and existing as a stable globular shape in an aqueous solution |  |
| 84 | EPS-Glc | *Phellinus vaninii* | Fermentation broth | Ethanol precipitation | Sepharose CL-6B chromatography | 6.255 × 10^5^ g/mol | Xyl:GlcA:Gal:Glc:Man:GalA = 1.52:19.53:2.39:24.28:30.68:21.60 | Existing as nearly globular shape forms in an aqueous solution | Xu et al., 2017 |
| 85 | EPS-Fru |  |  |  |  | 3.132 × 10^5^ g/mol | Rha:Rib:Xyl:GlcA:Gal:Glc:Man:GalA = 1.42:1.22:2.05:35.87:2.77:7.77:42.27:6.63 |  |  |
| 86 | EPS-Suc |  |  |  |  | 2.469 × 10^5^ g/mol | Rib:Xyl:GlcA:Gal:Glc:Man:GalA = 0.92:1.84:20.89:10.09:14.79:36.15:15.32 |  |  |
| 87 | Not given | *Phellinus igniarius* | Fruiting bodies | Ultrasonication, hot water treatment, and ethanol precipitation | ND | ND | Gal:Glc:Xyl:Man = 35.23:16.11:3.12:1.00 | ND | Ying et al., 2017 |
| 88 | Not given |  | Mycelia on 3 months |  |  |  | Gal:Glc:Man = 4.09:3.81:1.00 |  |  |
| 89 | Not given |  | Mycelia on 6 months |  |  |  | Gal:Glc:Man = 4.11:4.71:1.00 |  |  |
| 90 | PRP-S16 | *Phellinus ribis* | Fruiting bodies | Hot water extraction and ethanol precipitation | DEAE-Cellulose chromatography, Superdex 30 chromatography, chloro-sulfonic acid and formamide treatment, and DEAE-Sepharose Fast Flow chromatography | 18.3 kDa | ND | Existence of sulfate groups | Liu et al., 2018 |
| 91 | PIP-1 | *Phellinus igniarius* | Mycelia | Hot water extraction and ethanol precipitation | DEAE-Sepharose Fast Flow chromatography and Sepharose CL-4B chromatography | 812 kDa | Man:Glc:Gal = 2.41:87.74:3.86 | Containing a linear repeating backbone composed of Glc*p*, Gal*p*, and Man*p* joined by *α*-(1→4), *α*-(1→3), and *α*-(1→6) linkages, and single *α*-terminal-_D_-Glc*p* as side chains 6-*O*-linked to →4,6)-*α*-_D_-Glc*p*(1→ residues of the main chain | Yuan et al., 2018 |
| 92 | CK | *Phellinus igniarius* | Mycelia | Hot water extraction and ethanol precipitation | ND | 2.54 × 10^4^ and 3.8 kDa | Glc:Man:Rha = 9.0:1.0:3.0 | Containing *β*-configurations | Zhang et al., 2018 |
| 93 | JZx |  |  | Mutagenization, hot water extraction, and ethanol precipitation |  | 2.46 × 10^4^ and 1.5 kDa | Glc:Man:Rha = 2.0:1.0:16.0 |  |  |
| 94 | Not given | *Phellinus igniarius* | Mycelia | Hot water extraction and ethanol precipitation | ND | ND | ND | ND | Li et al., 2019 |
| 95 | IHSFP-1 | *Inonotus hispidus* | Mycelia | Ultrasonication and ethanol precipitation | DEAE-52 Cellulose chromatography and Sephadex G-200 gel chromatography | ND | ND | ND | Liu et al., 2019a |
| 96 | IHSFP-2 |  |  |  |  | 14.44 kDa | Glc:Man:Gal:GlcA:GalN:Ara:Rib = 51:21:20:3:2:2:1 | Containing _D_-pyranoses |  |
| 97 | IHSFP | *Inonotus hispidus* | Fermentation broth | Ethanol precipitation | DEAE-52 Cellulose chromatography and Sephadex G-200 gel chromatography | ND | Glc:Rha:Ara:Man:Gal:GlcN = 96.19:0.53:0.73:0.56:1.75:0.25 | Backbone composed of *α*-_D_-Glc*p* | Liu et al., 2019b |
| 98 | SHP-2 | *Sanghuangporus sanghuang* | Fermentation broth | Ethanol precipitation | DEAE-52 Cellulose chromatography and Sepharose CL-6B chromatography | 160 kDa | ND | Backbone composed of →4)-*β*-Man*p*-(1→4)-*α*-Ara*f*-(1→3,4)-*α*-Glc*p*(1→3,4)-*α*-Glc*p*-(1→3,4)-*α*-Glc*p*-(1→3,4)-*α*-Glc*p*-(1→3,4)-*α*-Glc*p*-(1→6)-*α*-Gal*p*-(1→4)-*β*-Man*p*-(1→, with five branches including four *α*-_D_-Glc*p*-(1→ and one *α*-_D_-Man*p*-(1→ | Ma et al., 2019 |
| 99 | Not given | *Phellinus igniarius* | Fruiting bodies | Enzyme-catalyzed extraction | ND | ND | ND | ND | Shi et al., 2019 |
| 100 | PPI | *Phellinus igniarius* | Fruiting bodies | Hot water extraction and ethanol precipitation | ND | ND | ND | ND | Wang L. et al., 2019 |
| 101 |  |  |  |  |  | 12.668 kDa | ND | ND | Wang Y. Q. et al., 2019 |
| 102 | Not given | *Phellinus baumii* | Fermentation broth | TPP with *t*-butanol as organic phase | ND | ND | ND | ND | Wang et al., 2019a |
| 103 | EPS |  |  | TPP with *t*-butanol as organic phase and ultrasonication |  | 231.3 and 41.93 (64.62%) kDa | Ara:Man:Glc:Gal = 2.4:29.3:3.9:1.0 | Containing *α*-configurations |  |
| 104 | EPS-C |  |  | Ethanol precipitation |  | 636.1 and 55.86 (87.03%) kDa | Ara:Man:Glc:Gal = 12.9:36.9:1.4:1.0 |  |  |
| 105 | EPS-D | *Phellinus baumii* | Fermentation broth | TPP with DMC as organic phase | ND | 1.589 × 10^3^ and 161 (96.1%) kDa | Ara:Man:Glc:Gal = 1.4:16.5:5.3:1.0 | Containing *α*-configurations | Wang et al., 2019b |
| 106 | EPS-T |  |  | TPP with *t*-butanol as organic phase |  | 231.3 and 41.9 (64.6%) kDa | Ara:Man:Glc:Gal = 2.4:29.3:2.2:1.0 |  |  |
| 107 | DPRG | *Phellinus ribis* | Fruiting bodies | Hot water extraction and ethanol precipitation | DEAE-Cellulose chromatography, Superdex 30 gel chromatography, degradation using H_2_O_2_-AA, and Sephacryl S-100 chromatography | 3.06 kDa | Glc | Containing *β*-configurations | Yang et al., 2019 |
| 108 | Not given | *Phellinus igniarius* | Fruiting bodies | Ultrasound combined with microwave treatment, hot water extraction, and ethanol precipitation | ND | ND | ND | ND | Ying et al., 2019 |
| 109 | PPB-2 | *Phellinus baumii* | Fruiting bodies | Hot water extraction, acetic acid treatment, ethanol precipitation, and ultrasonication | DEAE-52 Cellulose chromatography and Sephadex G-100 chromatography | 28.64 kDa | Fuc:Ara:Gal:Glc:Xyl:Man = 2.19:1.27:5.85:43.22:2.73:4.18 | ND | Zhang et al., 2019 |
| 110 | PL-N1 | *Phellinus linteus* | Mycelia | Hot water extraction, (NH_4_)_2_C_2_O_4_ treatment, NaOH/NaBH_4_ treatment, and ethanol precipitation | DEAE-Sephadex A-25 chromatography | 3.43 × 10^5^ kDa | Ara:Xyl:Glc:Gal = 4.0:6.7:1.3:1.0 | Backbone composed of (1→4)-linked *β*-_D_-Xyl*p* residues, (1→2)-linked *α*-_D_-Xyl*p* residues, (1→4)-linked *α*-_D_-Glc*p* residues, (1→5)-linked *β*-_D_-Ara*f* residues, and (1→4)-linked *β*-_D_-Xyl*p* residues | Chen et al., 2020 |
| 111 | SSEPS2 | *Sanghuangporus sanghuang* | Fermentation broth | Ethanol precipitation | DEAE-Sepharose Fast Flow chromatography and High-Resolution Sephacryl S-100 gel chromatography | 9.43 × 10^4^ g/mol | Man | Backbone composed of 1,3-linked and 1,2-linked *α*-_D_-Man*p*, with substitution at *O*-6 position of 1,2-linked *α*-_D_-Man*p* by 1,6-linked *α*-_D_-Man*p* residues and terminal *α*-_D_-Man*p* residues | Cheng et al., 2020a |
| 112 | SSIPS1 | *Sanghuangporus sanghuang* | Mycelia | Hot water extraction and ethanol precipitation | DEAE-Sepharose Fast Flow chromatography and High-Resolution Sephacryl S-100 gel chromatography | 2.35 × 10^4^ g/mol | Glc:Gal = 94.8:5.2 | Backbone composed of a 1,4-linked *α*-_D_-Glc*p* residue, with two branches at *O*-6 position consisting of 1,4-linked *α*-_D_-Glc*p* terminated with *α*-_D_-Glc*p*, and 1,4-linked *α*-_D_-Glc*p* and 1,4-linked *β*-Gal*p* terminated by *α*-_D_-Glc*p* | Cheng et al., 2020b |
| 113 | Not given | *Phellinus igniarius* | Fruiting bodies | Hot water extraction and ethanol precipitation | Sepharose CL-4B chromatography | ND | ND | ND | Hu et al., 2020 |
| 114 | SVP | *Sanghuangporus vaninii* | Fruiting bodies | Hot water extraction and ethanol precipitation | DEAE-52 Cellulose chromatography and Sephadex G-200 chromatography | 31.56 kDa | Man:Rha:GlcA:GalA:GlcN:Glc:GalN:Gal:Xyl:Ara:Fuc = 1.63:0.04:0.36:0.03:0.13:8.39:0.08:1.08:0.25:1.07:0.40 | ND | Wan et al., 2020 |
| 115 | PL-N | *Phellinus linteus* | Mycelia | Hot water extraction, (NH_4_)_2_C_2_O_4_ treatment, NaOH/NaBH_4_ treatment, and ethanol precipitation | ND | ND | Ara:Xyl:Glc:Gal = 5.5:7.8:1.8:1 | ND | Wang et al., 2020 |
| 116 | Not given | *Phellinus igniarius* | Mycelia | Hot water extraction and ethanol precipitation | ND | ND | ND | ND | Zhong, 2020 |
| 117 | Not given | *Sanghuangporus vaninii* | Fruiting bodies | Ultrasonication and ethanol precipitation | ND | ND | ND | ND | Chang et al., 2021 |
| 118 | Not given |  |  | Microwave extraction and ethanol precipitation |  |  |  |  |  |
| 119 | Not given |  |  | Heating reflux extraction and ethanol precipitation |  |  |  |  |  |
| 120 | Not given |  |  | Hot water extraction and ethanol precipitation |  |  |  |  |  |
| 121 | PSeP | *Phellinus igniarius* | Mycelia | Hot water extraction and ethanol precipitation | DEAE-52 Cellulose chromatography | 3.212 × 10^3^ kDa | Man:Rib:GlcA:Glc:Gal:Ara = 1:0.15:0.45:1.87:2.11:0.05 | Containing *α*- and *β*-anomeric configurations | Luo et al., 2021 |
| 122 | SHPS-1 | *Phellinus baumii* | Fruiting bodies | Hot water extraction and ethanol precipitation | DEAE-Sepharose Fast Flow chromatography and Sephadex G-200 gel-permeation chromatography | 46 kDa | Ara:Man:Glc:Gal = 2.2:15.7:49.3:32.8 | Backbone composed of 1,3-linked *β*-_D_-Glc*p* and 1,6-linked *α*-_D_-Gal*p* residues, with oligosaccharidic side chains of Ara*f*, Man*p*, and Gal*p* units at *C*-6 position of some Glc*p*s | Sun et al., 2021 |
| 123 | SePSP | *Sanghuangporus lonicericola* | Fermentation broth | Ethanol precipitation | EzLoad 16/10 DEAE Fast Flow chromatography and EzLoad 16/60 Chromdex 75 pg chromatography | ND | ND | ND | Zuo et al., 2021 |
| 124 | PLPS | *Phellinus linteus* | Mycelia | Hot water extraction and ATPS | ND | 6.93 × 10^5^, 5.22 × 10^4^, and 15.2 kDa | Ara:Xyl:Man:Glc:Gal = 1.0:1.8:3.8:40.1:1.4 | ND | Wu et al., 2022 |
| 125 | C-PIPS |  |  | Hot water extraction and ethanol precipitation |  | 5.84 × 10^5^, 6.73 × 10^4^, and 13.5 kDa | Ara:Xyl:Man:Glc:Gal = 1.0:1.5:3.4:25.2:1.1 |  |  |

***Abbreviations***: Mw, molecular weight; Ara, arabinose; Ara*f*, arabinofuranosyl; Fuc, fucose; Fuc*p*, fucopyranosyl; Fru, fructose; Gal, galactose; GalA, galacturonic acid; Gal*p*, galactopyranosyl; Glc, glucose; GlcA, glucuronic acid; GlcN, glucosamine; Glc*p*, glucopyranosyl; Man, mannose; Man*p*, mannopyranosyl; Rha, rhamnose; Rib, ribose; Suc, sucrose; Xyl, xylose; Xyl*p*, xylopyranosyl; TPP, three-phase partitioning; DMC, dimethyl carbonate; ATPS, aqueous two-phase system; AA, ascorbic acid; ND, not detected.

***References***

Baker, J. R., Kim, J. S., and Park, S. Y. (2008). Composition and proposed structure of a water-soluble glycan from the Keumsa Sangwhang Mushroom (*Phellinus linteus*). *Fitoterapia* 79, 345−350. doi: 10.1016/j.fitote.2008.03.002

Cao, C. L., Peng, F., and Cui, B. K. (2013). Chemical characterization and structure of exopolysaccharides from submerged culture of new medicinal mushroom from China, *Phellinus mori* (higher basidiomycetes). *Int. J. Med. Mushrooms* 15, 57−69. doi: 10.1615/IntJMedMushr.v15.i1.70

Chang, C., Zhao, J. H., Yu, W. J., Chen, Q. H., Qin, L. W., Wu, X. L., et al. (2021). Extraction technology and determination of polysaccharide from *Sanghuangporus vaninii*. *Chem. Reag.* 43, 973−978. doi: 10.13822 /j.cnki.hxsj.2021007987

Chen, C., Liu, X., Qi, S. S., Dias, A. C. P., Yan, J. K., and Zhang, X. Y. (2020). Hepatoprotective effect of *Phellinus linteus* mycelia polysaccharide (PL-N1) against acetaminophen-induced liver injury in mouse. *Int. J. Biol. Macromol.* 154, 1276−1284. doi: 10.1016/j.ijbiomac.2019.11.002

Chen, L., Pan, J. Z., Li, X., Zhou, Y., Meng, Q. L., and Wang, Q. (2011). Endo-polysaccharide of *Phellinus igniarius* exhibited anti-tumor effect through enhancement of cell mediated immunity. *Int. Immunopharmacol.* 11, 255−259. doi: 10.1016/j.intimp.2010.11.033

Cheng, J. W., Song, J. L., Liu, Y., Lu, N., Wang, Y. B., Hu, C. J., et al. (2020a). Conformational properties and biological activities of *α*-_D_-mannan from *Sanghuangporus sanghuang* in liquid culture. *Int. J. Biol. Macromol.* 164, 3568−3579. doi: 10.1016/j.ijbiomac.2020.08.112

Cheng, J. W., Song, J. L., Wei, H. L., Wang, Y. B., Huang, X. B., Liu, Y., et al. (2020b). Structural characterization and hypoglycemic activity of an intracellular polysaccharide from *Sanghuangporus sanghuang* mycelia. *Int. J. Biol. Macromol.* 164, 3305−3314. doi: 10.1016/j.ijbiomac.2020.08.202

Cheng, W., Qin, J. Z., Du, J. G., and Zhang, C. H. (2012). Optimization of ultrasonic-assisted enzymatic extraction of polysaccharide from *Phellinus igniarius*. *Mod. Food Sci. Technol.* 28, 662−666. doi: 10.3390/molecules24010147

Gao, W. W., Wang, W. D., Sun, W. J., Wang, M. F., Zhang, N., and Yu, S. W. (2017). Antitumor and immunomodulating activities of six *Phellinus igniarius* polysaccharides of different origins. *Exp. Ther. Med.* 14, 4627−4632. doi: 10.3892/etm.2017.5191

Ge, Q., Mao, J. W., Zhang, A. Q., Wang, Y. J., and Sun, P. L. (2013). Purification, chemical characterization, and antioxidant activity of a polysaccharide from the fruiting bodies of sanghuang mushroom (*Phellinus baumii* Pilát). *Food Sci. Biotechnol.* 22, 301−307. doi: 10.1007/s10068-013-0081-1

Ge, Q., Zhang, A. Q., and Sun, P. L. (2009a). Purification and structural elucidation of a novel fucoglucan from the fruiting bodies of *Phellinus baumii* Pilát. *J. Sci. Food Agr.* 89, 343−348. doi: 10.1002/jsfa.3464

Ge, Q., Zhang, A. Q., and Sun, P. L. (2009b). Structural investigation of a novel water-soluble heteropolysaccharide from the fruiting bodies of *Phellinus baumii* Pilát. *Food Chem.* 114, 391−395. doi: 10.1016/j.foodchem.2008.09.010

Guo, X., Zou, X., and Sun, M. (2010). Optimization of extraction process by response surface methodology and preliminary characterization of polysaccharides from *Phellinus igniarius*. *Carbohyd. Polym.* 80, 344−349. doi: 10.1016/j.carbpol.2009.11.028

He, P. X., Geng, L. J., Wang, J. Z., Wang, Z., Mao, D. B., and Xu, C. P. (2012). Purification, characterization and bioactivity of an extracellular polysaccharide produced from *Phellinus igniarius*. *Ann. Microbiol.* 62, 1697−1707. doi: 10.1007/s13213-012-0427-6

Hu, X. T., Ye, Y. J., Shi, G., Zhao, N. X., Gu, M. L., Yan, Y. N., et al. (2020). Extraction of polysaccharides from fruiting bodies of *Phellinus igniarius* and its protective effect on _D_-galactose induced 3T3 cell injury. *Food Sci.* 41, 204−211. doi: 10.7506/spkx1002-6630-20190906-078

Hwang, H. J., Kim, S. W., Choi, J. W., and Yun, J. W. (2003). Production and characterization of exopolysaccharides from submerged culture of *Phellinus linteus* KCTC 6190. *Enzyme Microb. Technol.* 33, 309−319. doi: 10.1016/S0141-0229(03)00131-5

Jia, X. W., Gao, M. Q., Li, M. Z., Wu, Y., Zeng, Y., and Xu, C. P. (2017). Molecular characterization of two polysaccharides from *Phellinus vaninii* Ljup and their cytotoxicity to cancer cell lines. *Anti-Cancer Age. Med. Chem.* 17, 1−8. doi: 10.2174/1871520617666170912144956

Jiang, P., Yuan, L., Cai, D. L., Jiao, L. L., and Zhang, L. P. (2015). Characterization and antioxidant activities of the polysaccharides from mycelium of *Phellinus pini* and culture medium. *Carbohyd. Polym.* 117, 600−604. doi: 10.1016/j.carbpol.2014.10.013

Jiang, P., Yuan, L., Huang, G. H., Wang, X. L., Li, X., Jiao, L. L., et al. (2016). Structural properties and immunoenhancement of an exopolysaccharide produced by *Phellinus pini*. *Int. J. Biol. Macromol.* 93, 566−571. doi: 10.1016/j.ijbiomac.2016.09.020

Jin, Q. L., Zhang, Z. F., Lv, G. Y., Cai, W. M., Cheng, J. W., Wang, J. G., et al. (2016). Antioxidant and DNA damage protecting potentials of polysaccharide extracted from *Phellinus baumii* using a delignification method. *Carbohyd. Polym.* 152, 575−582. doi: 10.1016/j.carbpol.2016.07.027

Kim, G. Y., Lee, J. Y., Lee, J. O., Ryu, C. H., Choi, B. T., Jeong, Y. K., et al. (2006). Partial characterization and immunostimulatory effect of a novel polysaccharide-protein complex extracted from *Phellinus linteus*. *Biosci. Biotechnol. Biochem.* 70, 1218−1226. doi: 10.1271/bbb.70.1218

Kim, G. Y., Park, H. S., Nam, B. H., Lee, S. J., and Lee, J. D. (2003). Purification and characterization of acidic proteo-heteroglycan from the fruiting body of *Phellinus linteus* (Berk. & M.A. Curtis) Teng. *Bioresour. Technol.* 89, 81−87. doi: 10.1016/S0960-8524(02)00273-0

Kim, H. M., Kang, J. S., Kim, J. Y., Park, S. K., Kim, H. S., Lee, Y. J., et al. (2010). Evaluation of antidiabetic activity of polysaccharide isolated from *Phellinus linteus* in non-obese diabetic mouse. *Int. Immunopharmacol.* 10, 72−78. doi: 10.1016/j.intimp.2009.09.024

Lee, S. M., Kim, S. M., Lee, Y. H., Kim, W. J., Park, J. K., Park, Y. I., et al. (2010). Macromolecules isolated from *Phellinus pini* fruiting body: Chemical characterization and antiviral activity. *Macromol. Res.* 18, 602−609. doi: 10.1007/s13233-010-0615-9

Li, R. X., Wang, Y. T., Xia, J. F., Luo, D. Q., and Wang, T. C. (2019). Optimization of extraction process of polysaccharide of *Phellinus igniarius* mycelium and analysis of its antioxidant activity *in vitro*. *Chin. Agr. Sci. Bull.* 35, 143−150.

Li, S. C., Yang, X. M., Ma, H. L., Yan, J. K., and Guo, D. Z. (2015). Purification, characterization and antitumor activity of polysaccharides extracted from *Phellinus igniarius* mycelia. *Carbohyd. Polym.* 133, 24−30. doi: 10.1016/j.carbpol.2015.07.013

Li, T. T., Yang, Y., Liu, Y. F., Zhou, S., Yan, M. Q., Wu, D., et al. (2015). Physicochemical characteristics and biological activities of polysaccharide fractions from *Phellinus baumii* cultured with different methods. *Int. J. Biol. Macromol.* 81, 1082−1088. doi: 10.1016/j.ijbiomac.2015.09.001

Li, X., Jiao, L. L., Zhang, X., Tian, W. M., Chen, S., and Zhang, L. P. (2008). Anti-tumor and immunomodulating activities of proteoglycans from mycelium of *Phellinus nigricans* and culture medium. *Int. Immunopharmacol.* 8, 909−915. doi: 10.1016/j.intimp.2008.02.008

Liu, M. M., Zeng, P., Li, X. T., and Shi, L. G. (2016). Antitumor and immunomodulation activities of polysaccharide from *Phellinus baumii*. *Int. J. Biol. Macromol.* 91, 1199−1205. doi: 10.1016/j.ijbiomac.2016.06.086

Liu, X., Hou, R. L., Xu, K. Q., Chen, L., Wu, X. P., Lin, W. X., et al. (2019a). Extraction, characterization and antioxidant activity analysis of the polysaccharide from the solid-state fermentation substrate of *Inonotus hispidus*. *Int. J. Biol. Macromol.* 123, 468−476. doi: 10.1016/j.ijbiomac.2018.11.069

Liu, X., Hou, R. L., Yan, J. J., Xu, K. Q., Wu, X. P., Lin, W. X., et al. (2019b). Purification and characterization of *Inonotus hispidus* exopolysaccharide and its protective effect on acute alcoholic liver injury in mice. *Int. J. Biol. Macromol.* 129, 41−49. doi: 10.1016/j.ijbiomac.2019.02.011

Liu, Y. H., Liu, C. H., Jiang, H. Q., Zhou, H. L., Li, P. L., and Wang, F. S. (2015). Isolation, structural characterization and neurotrophic activity of a polysaccharide from *Phellinus ribis*. *Carbohyd. Polym.* 127, 145−151. doi: 10.1016/j.carbpol.2015.03.057

Liu, Y. H., and Wang, F. S. (2007). Structural characterization of an active polysaccharide from *Phellinus ribis*. *Carbohyd. Polym.* 70, 386−392. doi: 10.1016/j.carbpol.2007.04.019

Liu, Y. H., Xu, J. Z., Zong, A. Z., Wang, J. H., Liu, Y. G., Jia, W., et al. (2018). Anti-angiogenic activity and mechanism of a chemically sulfated natural glucan from *Phellinus ribis*. *Int. J. Biol. Macromol.* 107, 2475−2483. doi: 10.1016/j.ijbiomac.2017.10.134

Luo, J. G., Liu, J., Ke, C. L., Qiao, D. L., Ye, H., Sun, Y., et al. (2009). Optimization of medium composition for the production of exopolysaccharides from *Phellinus baumii* Pilát in submerged culture and the immuno-stimulating activity of exopolysaccharides. *Carbohyd. Polym.* 78, 409−415. doi: 10.1016/j.carbpol.2009.04.038

Luo, J. G., Liu, J., Sun, Y., Ye, H., Zhou, C. H., and Zeng, X. X. (2010). Medium optimization, preliminary characterization and antioxidant activity *in vivo* of mycelial polysaccharide from *Phellinus baumii* Pilát. *Carbohyd. Polym.* 81, 533−540. doi: 10.1016/j.carbpol.2010.03.010

Luo, L. J., Wang, Y. X., Zhang, S., Guo, L., Jia, G. T., Lin, W. P., et al. (2021). Preparation and characterization of selenium-rich polysaccharide from *Phellinus igniarius* and its effects on wound healing. *Carbohyd. Polym.* 264, 117982. doi: 10.1016/j.carbpol.2021.117982

Ma, X. K., Guo, D. D., Peterson, E. C., Dun, Y., and Li, D. Y. (2016). Structural characterization and anti-aging activity of a novel extracellular polysaccharide from fungus *Phellinus* sp. in mammalian system. *Food Funct.* 7, 3468−3479. doi: 10.1039/C6FO00422A

Ma, X. K., She, X., Peterson, E. C., Wang, Y. Z., Zheng, P., Ma, H. Y., et al. (2019). A newly characterized exopolysaccharide from *Sanghuangporus sanghuang*. *Int. J. Biol. Macromol.* 57, 812−820. doi: 10.1007/s12275-019-9036-4

Mei, Y. X., Zhu, H., Hu, Q. M., Liu, Y. Y., Zhao, S. M., Peng, N., et al. (2015). A novel polysaccharide from mycelia of cultured *Phellinus linteus* displays antitumor activity through apoptosis. *Carbohyd. Polym.* 124, 90−97. doi: 10.1016/j.carbpol.2015.02.009

Pei, J. J., Wang, Z. B., Ma, H. L., and Yan, J. K. (2015). Structural features and antitumor activity of a novel polysaccharide from alkaline extract of *Phellinus linteus* mycelia. *Carbohyd. Polym.* 115, 472−477. doi: 10.1016/j.carbpol.2014.09.017

Shi, Y. B., Bai, W. D., Zhao, W. H., Qian, M., and Bai, Y. L. (2019). Optimization of enzymatic hydrolysis-assisted extraction of polysaccharides from *Phellinus igniarius*. *Farm Prod. Process.* 9, 29−32+35. doi: 10.16693/j.cnki.1671-9646(X).2019.09.042

Shon, Y. H., and Nam, K. S. (2002). Cancer chemoprevention: Inhibitory effect of soybeans fermented with basidiomycetes on 7,12-dimethylbenz[a]anthracene/12-*O*-tetradecanoylphorbol-13-acetate-induced mouse skin carcinogenesis. *Biotechnol. Lett.* 24, 1005−1010.

Shon, Y. H., and Nam, K. S. (2003). Inhibition of cytochrome P450 isozymes in rat liver microsomes by polysaccharides derived from *Phellinus linteus*. *Biotechnol. Lett.* 25, 167−172. doi: 10.1016/S0308-8146(03)00015-3

Suabjakyong, P., Nishimura, K., Toida, T., and van Griensven, L. J. L. D. (2015). Structural characterization and immunomodulatory effects of polysaccharides from *Phellinus linteus* and *Phellinus igniarius* on the IL-6/IL-10 cytokine balance of the mouse macrophage cell lines (RAW264.7). *Food Funct.* 6, 2834−2844. doi: 10.1039/c5fo00491h

Sun, Y. Q., Huo, J. X., Zhong, S., Zhu, J. X., Li, Y. G., and Li, X. J. (2021). Chemical structure and anti-inflammatory activity of a branched polysaccharide isolated from *Phellinus baumii*. *Carbohyd. Polym.* 268, 118214. doi: 10.1016/j.carbpol.2021.118214

van Griensven, L. J. L. D., and Verhoeven, H. A. (2013). *Phellinus linteus* polysaccharide extracts increase the mitochondrial membrane potential and cause apoptotic death of THP-1 monocytes. *Chin. Med.* 8, 25. doi: 10.1186/1749-8546-8-25

Wan, X. L., Jin, X., Xie, M. L., Liu, J., Gontcharov, A. A., Wang, H., et al. (2020). Characterization of a polysaccharide from *Sanghuangporus vaninii* and its antitumor regulation via activation of the p53 signaling pathway in breast cancer MCF-7 cells. *Int. J. Biol. Macromol.* 163, 865−877. doi: 10.1016/j.ijbiomac.2020.06.279

Wang, K., Ding, Z. C., Pei, J. J., and Yan, J. K. (2020). Antioxidant activities of polysaccharides from *Phellinus linteus* mycelia by alkaline extraction. *Sci. Technol. Food Ind.* 41, 289−294. doi: 10.13386/j.issn1002-0306.2020.01.047

Wang, L., Yao, L., Jin, Y. W., Jin, C. Y., Dong, Y., Shou, D., et al. (2019). Activation effect of human TLR4 signaling pathway by polysaccharide from *Phellinus igniarius*. *Chin. J. Mod. Appl. Pharm.* 36, 1178−1182. doi: 10.13748/j.cnki.issn1007-7693.2019.10.002

Wang, Y. Q., Mao, J. B., Zhou, M. Q., Jin, Y. W., Lou, C. H., Dong, Y., et al. (2019). Polysaccharide from *Phellinus igniarius* activates TLR4-mediated signaling pathways in macrophages and shows immune adjuvant activity in mice. *Int. J. Biol. Macromol.* 123, 157−166. doi: 10.1016/j.ijbiomac.2018.11.066

Wang, Y. Y., Ma, H. L., Ding, Z. C., Yang, Y., Wang, W. H., Zhang, H. N., et al. (2019a). Three-phase partitioning for the direct extraction and separation of bioactive exopolysaccharides from the cultured broth of *Phellinus baumii*. *Int. J. Biol. Macromol.* 123, 201−209. doi: 10.1016/j.ijbiomac.2018.11.065

Wang, Y. Y., Ma, H. L., Yan, J. K., Wang, K. D., Yang, Y., Wang, W. H., et al. (2019b). Three-phase partitioning system with dimethyl carbonate as organic phase for partitioning of exopolysaccharides from *Phellinus baumii*. *Int. J. Biol. Macromol.* 131, 941−948. doi: 10.1016/j.ijbiomac.2019.03.149

Wang, Z. B., Pei, J. J., Ma, H. L., Cai, P. F., and Yan, J. K. (2014). Effect of extraction media on preliminary characterizations and antioxidant activities of *Phellinus linteus* polysaccharides. *Carbohyd. Polym.* 109, 49−55. doi: 10.1016/j.carbpol.2014.03.057

Wang, Z. Y., Wang, C. Y., and Quan, Y. (2014a). Extraction of polysaccharides from *Phellinus nigricans* mycelia and their antioxidant activities *in vitro*. *Carbohyd. Polym.* 99, 110−115. doi: 10.1016/j.carbpol.2013.08.073

Wang, Z. Y., Zhou, F., and Quan, Y. (2014b). Antioxidant and immunological activity *in vitro* of polysaccharides from *Phellinus nigricans* mycelia. *Int. J. Biol. Macromol.* 64, 139−143. doi: 10.1016/j.ijbiomac.2013.11.038

Wu, S. J., Liaw, C. C., Pan, S. Z., Yang, H. C., and Ng, L. T. (2013). *Phellinus linteus* polysaccharides and their immunomodulatory properties in human monocytic cells. *J. Funct. Foods* 5, 679−688. doi: 10.1016/j.jff.2013.01.011

Wu, Y., Liu, H., Li, Z. H., Huang, D. Y., Nong, L. Z., Ning, Z. X., et al. (2022). Purification of polysaccharides from *Phellinus linteus* by using an aqueous two-phase system and evaluation of the physicochemical and antioxidant properties of polysaccharides *in vitro*. *Prep. Biochem. Biotechnol.* 52, 89−98. doi: 10.1080/10826068.2021.1911815

Xu, C. P., Yu, J. W., Zhao, S. S., Wu, S. S., He, P. X., Jia, X. W., et al. (2017). Effect of carbon source on production, characterization and bioactivity of exopolysaccharide produced by *Phellinus vaninii* Ljup. *An. Acad. Bras. Ciênc.* 89, 2033−2041. doi: 10.1590/0001-3765201720150786

Xu, Y., Zhao, X. Y., Cao, H., Sheng, S., Wang, J., and Wu, F. A. (2016). Enzyme-catalyzed extraction and antioxidant activity of polysaccharides from *Phellinus igniarius*. *Curr. Top. Nutraceut. Res.* 14, 171−180.

Xue, Q., Sun, J., Zhao, M. W., Zhang, K. Y., and Lai, R. (2011). Immunostimulatory and anti-tumor activity of a water-soluble polysaccharide from *Phellinus baumii* mycelia. *World J. Microbiol. Biotechnol.* 27, 1017−1023. doi: 10.1007/s11274-010-0545-x

Yan, J. K., Wang, Y. Y., Ma, H. L., and Wang, Z. B. (2016a). Ultrasonic effects on the degradation kinetics, preliminary characterization and antioxidant activities of polysaccharides from *Phellinus linteus* mycelia. *Ultrason. Sonochem.* 29, 251−257. doi: 10.1016/j.ultsonch.2015.10.005

Yan, J. K., Wang, Y. Y., Ma, H. L., Wang, Z. B., and Pei, J. J. (2016b). Structural characteristics and antioxidant activity *in vivo* of a polysaccharide isolated from *Phellinus linteus* mycelia. *J. Taiwan Inst. Chem. Eng.* 65, 110−117. doi: 10.1016/j.jtice.2016.05.052

Yan, J. K., Wang, Y. Y., Wang, Z. B., Ma, H. L., Pei, J. J., and Wu, J. Y. (2016c). Structure and antioxidative property of a polysaccharide from an ammonium oxalate extract of *Phellinus linteus*. *Int. J. Biol. Macromol.* 91, 92−99. doi: 10.1016/j.ijbiomac.2016.05.063

Yang, P., Jin, J., Liu, Q., Ma, D. M., Li, J., Zhang, Y. Q., et al. (2019). Optimization of degradation conditions with PRG, a polysaccharide from *Phellinus ribis*, by RSM and the neuroprotective activity in PC12 cells damaged by A*β*_25-35_. *Molecules* 24, 3010. doi: 10.3390/molecules24163010

Yang, Y., Ye, L. B., Zhang, J. S., Liu, Y. F., and Tang, Q. J. (2009). Structural analysis of a bioactive polysaccharide, PISP1, from the medicinal mushroom *Phellinus igniarius*. *Biosci. Biotechnol. Biochem.* 73, 134−139. doi: 10.1271/bbb.80546

Yang, Y., Zhang, J. S., Liu, Y. F., Tang, Q. J., Zhao, Z. G., and Xia, W. S. (2007). Structural elucidation of a 3-*O*-methyl-_D_-galactose-containing neutral polysaccharide from the fruiting bodies of *Phellinus igniarius*. *Carbohyd. Res.* 342, 1063−1070. doi: 10.1016/j.carres.2007.02.019

Ying, R. F., Huang, M. G., Wang, Y. S., Wu, C. E., Li, T. T., and Fan, G. J. (2019). Ultrasonic-microwave synergistic assisted extraction and activity of polysaccharides from *Phellinus igniarius*. *Food Res. Dev.* 40, 82−88. doi: 10.12161/j.issn.1005-6521.2019.21.015

Ying, R. F., Wu, C. E., Huang, M. G., and Wang, Y. S. (2017). Anti-tumor activity of polysaccharides from *Phellinus igniarius* fruiting body and mycelium. *China Food Addit.* 12, 57−61.

Yuan, Q. X., Zhao, L. Y., Li, Z. H., Harqin, C., Peng, Y. F., and Liu, J. K. (2018). Physicochemical analysis, structural elucidation and bioactivities of a high-molecular-weight polysaccharide from *Phellinus igniarius* mycelia. *Int. J. Biol. Macromol.* 120, 1855−1864. doi: 10.1016/j.ijbiomac.2018.09.192

Zhang, H. N., Ma, H. L., Liu, W., Pei, J. J., Wang, Z. B., Zhou, H. J., et al. (2014). Ultrasound enhanced production and antioxidant activity of polysaccharides from mycelial fermentation of *Phellinus igniarius*. *Carbohyd. Polym.* 113, 380−387. doi: 10.1016/j.carbpol.2014.07.027

Zhang, H. N., Ma, H. L., Zhou, C. S., Yan, Y., Yin, X. L., and Yan, J. K. (2018). Enhanced production and antioxidant activity of endo-polysaccharides from *Phellinus igniarius* mutants screened by low power He-Ne laser and ultraviolet induction. *Bioact. Carbohyd. Diet. Fibre* 15, 30−36. doi: 10.1016/j.bcdf.2016.11.006

Zhang, Z. F., Lv, G. Y., Cheng, J. W., Cai, W. M., Fan, L. F., and Miao, L. X. (2019). Characterization and biological activities of polysaccharides from artificially cultivated *Phellinus baumii*. *Int. J. Biol. Macromol.* 129, 861−868. doi: 10.1016/j.ijbiomac.2019.02.082

Zhang, Z. F., Lv, G. Y., Song, T. T., Jin, Q. L., Huang, J. B., Fan, L. F., et al. (2015). Comparison of the preliminary characterizations and antioxidant properties of polysaccharides obtained from *Phellinus baumii* growth on different culture substrates. *Carbohyd. Polym.* 132, 397−399. doi: 10.1016/j.carbpol.2015.06.006

Zhao, C., Liao, Z. S., Wu, X. Q., Liu, Y. L., Liu, X. Y., Lin, Z. X., et al. (2014). Isolation, purification, and structural features of a polysaccharide from *Phellinus linteus* and its hypoglycemic effect in alloxan-induced diabetic mice. *J. Food Sci.* 79, H1002−H1010. doi: 10.1111/1750-3841.12464

Zhong, C. (2020). Anti-fatigue effect of fermentation broth polysaccharose of *Phellinus igniarius*. *Edible Fungi China* 39, 46−48. doi: 10.13629/j.cnki.53-1054.2020.06.012

Zuo, K., Tang, K. J., Liang, Y., Xu, Y. F., Sheng, K. L., Kong, X. W., et al. (2021). Purification and antioxidant and anti-inflammatory activity of extracellular polysaccharopeptide from sanghuang mushroom, *Sanghuangporus lonicericola*. *J. Sci. Food Agr.* 101, 1009−1020. doi: 10.1002/jsfa.10709
