# Supplementary material for: Current advances and potential trends of the polysaccharides derived from medicinal mushrooms sanghuang
Source: Front Microbiol. 2022 Aug 3;13:965934. doi: 10.3389/fmicb.2022.965934 (PMC9382022; doi:10.3389/fmicb.2022.965934)
Supplement: Supplementary file 2 [file Table_2.doc]

**SUPPLEMENTARY TABLE 2.** Summary of the polysaccharides from different sanghuang species reported in recent years and their integrated information in terms of bioactivities, involved mechanisms, and structure-bioactivity relationships.

| **No.** | **Compound name** | **Bioactivity** | **Involved mechanism** | **Structure-bioactivity relationship** | **Reference** |
| --- | --- | --- | --- | --- | --- |
| 1 | Not given | Anti-tumor activity | Induction of phase II enzymes such as QR and GST | ND | Shon and Nam, 2002 |
| 2 | Fr-I | ND | ND | ND | Hwang et al., 2003 |
| 3 | Fr-II |  |  |  |  |
| 4 | Fr-III |  |  |  |  |
| 5 | Not given | Anti-tumor and immunostimulating activities | Increasing proliferation of whole spleen cells | Potent antitumor property was not the results of *α*- and *β*-glycosidic linkages, but rather 3D conformation, branching ration, and molecular complexity | Kim et al., 2003 |
| 6 | Not given | Anti-tumor activity | Inhibitory potency for activities of cytochrome P450 1A1, 1A2, 2B1, and 2E1 in rat liver microsomes to block the conversion of procarcinogens into ultimate carcinogens | ND | Shon and Nam, 2003 |
| 7 | Not given |  |  |  |  |
| 8 | PPC | Anti-tumor and Immunomodulatory activities | Inhibiting proliferation of cancer cells, increasing proliferation of B-lymphocytes, and production of cytokines and NO, as well as up-regulating natural killer cell activity | ND | Kim G. Y. et al., 2006 |
| 9 | PRP | Immunomodulatory activity | Stimulating proliferation of spleen lymphocytes | ND | Liu and Wang, 2007 |
| 10 | PIP60-1 | Immunomodulatory activity | Stimulating proliferation of spleen lymphocytes | ND | Yang et al., 2007 |
| 11 | Not given | ND | ND | A core *β*(1−3)-linked glucan chain with *β*(1−6) branch points was a common feature of mushroom glycans possessing anti-tumor activity | Baker et al., 2008 |
| 12 | PNM1 | Anti-tumor and immunomodulatory activities | Increasing expression of TNF-*α* and NO production as well as stimulating proliferation of lymphocytes | ND | Li et al., 2008 |
| 13 | PNW1 |  |  |  |  |
| 14 | PBF4 | ND | ND | ND | Ge et al., 2009a |
| 15 | PBF2 | ND | ND | ND | Ge et al., 2009b |
| 16 | EPS | Immunostimulating activity | Stimulating proliferation of splenocyte and acid phosphatase activity in peritoneal macrophages | ND | Luo et al., 2009 |
| 17 | PISP1 | Immunomodulatory activity | Stimulating proliferation of spleen lymphocytes | ND | Yang et al., 2009 |
| 18 | IPS | ND | ND | ND | Guo et al., 2010 |
| 19 | PLP | Anti-diabetic activity | Inhibiting expression of inflammatory cytokines including IFN-*γ*, IL-2, and TNF-*α* | ND | Kim et al., 2010 |
| 20 | EP-AV1 | Anti-viral activity | Inhibiting neuraminidase activity | ND | Lee S. M. et al., 2010 |
| 21 | EP-AV2 |  |  |  |  |
| 22 | PBMP | Antioxidant activity | Enhancing activities of SOD, CAT, and GSH-Px, and TEAC level, as well as decreasing MDA concentration | ND | Luo et al., 2010 |
| 23 | PIE | Anti-tumor and immunomodulatory activities | Inhibiting proliferation of cancer cells as well as promoting immune cells activities and cytokines secretion such as IL-2, IL-18, and TNF-*α* | ND | Chen et al., 2011 |
| 24 | PB | Anti-tumor and immunomodulatory activities | Increasing proliferation of macrophages, NO production, and cytokines expression, as well as inducing cell cycle arrest at *S* phase and apoptosis | ND | Xue et al., 2011 |
| 25 | Not given | ND | ND | ND | Cheng et al., 2012 |
| 26 | EPS | Antioxidant and anti-tumor activities | Scavenging DPPH, hydroxyl, and ABTS radicals, as well as inhibiting proliferation of cancer cells | Electron-withdrawing carboxyl groups substituted at *C*-5 position of the sugar residue could activate the hydrogen atom of sugar residues through field and inductive effects. The higher the activating capacity of group, the stronger the antioxidant activity of EPS. More expanded chains and the higher the water solubility could lead to enhanced antitumor activity | He et al., 2012 |
| 27 | PM-ESP1 | ND | ND | ND | Cao et al., 2013 |
| 28 | PM-ESP3 | Antioxidant activity | Ease of abstraction of anomeric hydrogen from internal monosaccharide unit |  |  |
| 29 | PBF3 | Antioxidant activity | Scavenging superoxide, hydroxyl, and DPPH radicals | ND | Ge et al., 2013 |
| 30 | Not given | Antioxidant and anti-tumor activities | Inhibiting ROS production, inducing apoptosis of human leukemia-derived monocytes THP-1, and increasing potential of mitochondrial membrane | ND | van Griensven and Verhoeven, 2013 |
| 31 | PSCPL | Immunomodulatory activity | Inhibiting ROS formation, down-regulating cytokines (TNF-*α*, IL-1*α*, IL-1*β*, and IL-4) production, VCAM-1 expression, and JNK and p38 activation and phosphorylation, as well as suppressing NF-*κ*B activation, MyD88-dependent, and MAPK signaling pathways | ND | Wu et al., 2013 |
| 32 | PL-A | Antioxidant activity | ND | Low Mw and high contents of carbohydrate and uronic acid of a polysaccharide owned stronger antioxidant activity | Wang Z. B. et al., 2014 |
| 33 | PL-N |  |  |  |  |
| 34 | PL-W |  |  |  |  |
| 35 | Not given | Antioxidant activity | Scavenging superoxide, hydroxyl, and DPPH radicals, as well as exhibiting stronger reducing power | ND | Wang et al., 2014a |
| 36 | PNMP 1 | Antioxidant and immunomodulatory activities | Scavenging superoxide, hydroxyl, and DPPH radicals, as well as increasing proliferation of lymphocytes | ND | Wang et al., 2014b |
| 37 | PNMP 2 |  |  |  |  |
| 38 | PNMP 3 |  |  |  |  |
| 39 | PIPS | Antioxidant activity | Ultrasound could change morphology and structure of mycelia, and accelerate transfer of nutrients and metabolites | Low Mw and higher amounts of hydroxyl groups and uronic acid of a polysaccharide exhibited higher antioxidant activity | Zhang et al., 2014 |
| 40 | C-PIPS |  | ND |  |  |
| 41 | PLP-I | Hypoglycemic activity | Regulating expression of inflammatory cytokines | The (1-4)-linked and/or (1-6)-linked residues in *β*-(1-6)-branched (1-3)-*β*-_D_-glucan were needed for hypoglycemic effect | Zhao et al., 2014 |
| 42 | PPM | Antioxidant and immunomodulatory activities | ND | A low-Mw polysaccharide had higher antioxidant activity | Jiang et al., 2015 |
| 43 | PPE | Macrophages-activating ability | Augmenting phagocytosis and enhancing production of NO, TNF-*α*, and ROS | High content of Man in a polysaccharide enhanced immunomodulatory activity | Jiang et al., 2015, 2016 |
| 44 | IPSW-1 | Anti-tumor activity | ND | High Mw, carboxylate groups and water solubility of a polysaccharide indicated higher antitumor activity | Li S. C. et al., 2015 |
| 45 | IPSW-2 |  |  |  |  |
| 46 | IPSW-3 |  |  |  |  |
| 47 | IPSW-4 |  |  |  |  |
| 48 | FP30 | Macrophage stimulatory activity | Improving NO production | A polysaccharide fraction derived from submerged mycelia exhibited higher macrophages stimulatory activity | Li T. T. et al., 2015 |
| 49 | FP50 |  |  |  |  |
| 50 | FP70 |  |  |  |  |
| 51 | LP30 |  |  |  |  |
| 52 | LP50 |  |  |  |  |
| 53 | LP70 |  |  |  |  |
| 54 | SP30 |  |  |  |  |
| 55 | SP50 |  |  |  |  |
| 56 | SP70 |  |  |  |  |
| 57 | PRG | Neurotrophic activity | Promoting neurite outgrowth of nerve growth factor-stimulated PC12 cells | ND | Liu et al., 2015 |
| 58 | PLPS-1 | Anti-tumor activity | Decreasing expression of anti-apoptotic Bcl-2 protein and increasing level of pro-apoptotic Bax protein | Striking differences in anti-tumor activities between PLPS-1 and PLPS-2 resulted from these structural differences | Mei et al., 2015 |
| 59 | PLPS-2 |  | ND |  |  |
| 60 | PL-N1 | Anti-tumor activity | Inhibiting proliferation of cancer cells | High Mw and water solubility of a polysaccharide exhibited higher antitumor activity | Pei et al., 2015 |
| 61 | Not given | Immunomodulatory activity | Decreasing TNF-*α* and IL-6 expression, increasing IL-10 expression, and restoring IL-6/IL-10 balance | ND | Suabjakyong et al., 2015 |
| 62 | Not given |  |  |  |  |
| 63 | PPB-MB | Antioxidant activity | Inhibiting liver peroxidation, scavenging ABTS radicals, and chelating Fe^2+^ | A polysaccharide with low Mw and high amount of uronic acid had higher antioxidant activity | Zhang et al., 2015 |
| 64 | PPB-MW |  |  |  |  |
| 65 | PPB-MM |  |  |  |  |
| 66 | PPB-2 | Antioxidant and DNA damage protecting activities | Scavenging DPPH, superoxide, and hydroxyl radicals | ND | Jin et al., 2016 |
| 67 | PPB | Anti-tumor and immunomodulatory activities | Inhibiting proliferation of cancer cells, blocking cell division in *G*_0_/*G*_1_ period, as well as promoting immune cells activities and cytokines secretion | ND | Liu et al., 2016 |
| 68 | SHP-1 | Antioxidant, anti-aging, anti-inflammatory, and hepatoprotective activities | Increasing activities of CAT and SOD, and TEAC and organ indices, reducing lipofuscin content, as well as ameliorating histopathological hepatic lesions and apoptosis | ND | Ma et al., 2016 |
| 69 | PIPs | Antioxidant activity | Exhibiting stronger reducing power and scavenging ABTS and DPPH radicals | ND | Xu et al., 2016 |
| 70 | PL-N | Antioxidant and immunomodulatory activities | Scavenging hydroxyl radicals and exhibiting stronger TEAC and FRAP | Low Mw and viscosity of a polysaccharide exhibited stronger antioxidant activity | Yan et al., 2016a |
| 71 | PL-N1 |  |  |  |  |
| 72 | PL-N2 |  |  |  |  |
| 73 | PL-N3 |  |  |  |  |
| 74 | PLP1-I | Antioxidant activity | Increasing viscera indices, and activities of SOD, GSH-Px, and CAT, as well as decreasing MDA concentration | ND | Yan et al., 2016b |
| 75 | PL-A11 | Antioxidant and immunomodulatory activities | Increasing viscera indices, and activities of SOD, GSH-Px, and CAT, as well as decreasing MDA concentration | ND | Yan et al., 2016c |
| 76 | DMPIP | Anti-tumor and immunomodulatory activities | Inhibiting proliferation of cancer cells and inducing production of IFN-*γ*, IL-12, and IL-2 | ND | Gao et al., 2017 |
| 77 | HPIP |  |  |  |  |
| 78 | GPIP |  |  |  |  |
| 79 | DWPIP |  |  |  |  |
| 80 | SPIP |  |  |  |  |
| 81 | UPIP |  |  |  |  |
| 82 | PV-B | Anti-tumor activity | Inhibiting proliferation of cancer cells | A *β*-glucan with good water solubility, relatively high chain stiffness, and moderate Mw exhibited antitumor activity | Jia et al., 2017 |
| 83 | PV-W |  |  |  |  |
| 84 | EPS-Glc | Antioxidant activity | Scavenging hydroxyl and DPPH radicals | Suc as a better carbon source from the viewpoint of antioxidant activity due to the relatively high content of Gal in EPS and moderate Mw | Xu et al., 2017 |
| 85 | EPS-Fru |  |  |  |  |
| 86 | EPS-Suc |  |  |  |  |
| 87 | Not given | Anti-tumor activity | Inhibiting proliferation of cancer cells | A polysaccharide of mycelium within short growing period exhibited stronger anti-tumor activity | Ying et al., 2017 |
| 88 | Not given |  |  |  |  |
| 89 | Not given |  |  |  |  |
| 90 | PRP-S16 | Anti-angiogenic activity | Inhibiting VEGF-induced signaling pathway | ND | Liu et al., 2018 |
| 91 | PIP-1 | Antioxidant and anti-tumor activities | Scavenging DPPH and hydroxyl radicals, and chelating Fe^2+^, as well as inhibiting proliferation of cancer cells | Low Mw of a polysaccharide and the more reducing ends, sulfate content or carboxylic groups exhibited stronger antioxidant activity | Yuan et al., 2018 |
| 92 | CK | Antioxidant activity | Exhibiting stronger TEAC and FRAP, and scavenging hydroxyl radicals | A polysaccharide fraction with low Mw exhibited stronger antioxidant activity | Zhang et al., 2018 |
| 93 | JZx |  |  |  |  |
| 94 | Not given | Antioxidant activity | Scavenging ABTS and DPPH radicals | ND | Li et al., 2019 |
| 95 | IHSFP-1 | ND | ND | ND | Liu et al., 2019a |
| 96 | IHSFP-2 | Antioxidant activity and protective effect | Scavenging ABTS, DPPH, and hydroxyl radicals, reducing intracellular ROS level, and inhibiting oxidative stress of cells |  |  |
| 97 | IHSFP | Liver protective activity | Activating Nrf2 signaling pathway and increasing expression of related antioxidant enzymes such as SOD, NQO1, and CAT | A *α*-glucan had many biological activities such as antioxidant, anti-tumor, and immunomodulatory | Liu et al., 2019b |
| 98 | SHP-2 | Antioxidant, anti-aging, anti-inflammatory, and hepatoprotective activities | Increasing activities of CAT and SOD, and TEAC and organ indices, reducing lipofuscin contents, as well as ameliorating histopathological hepatic lesions and apoptosis | A polysaccharide with more complex structure and larger Mw, involving more side-branches and residues, had different activities | Ma et al., 2019 |
| 99 | Not given | ND | ND | ND | Shi et al., 2019 |
| 100 | PPI | Immunomodulatory activity | Increasing mRNA expression of cytokines such as TNF-*α*, IL-6, IL-12, IL-1*β*, and COX-2 in MyD88 pathway, as well as IP-10 and IFN-*β* in TRIF pathway | ND | Wang L. et al., 2019 |
| 101 |  |  |  |  | Wang Y. Q. et al., 2019 |
| 102 | Not given | ND | ND | ND | Wang et al., 2019a |
| 103 | EPS | Antioxidant, anti-hyperglycemic, and immunomodulatory activities | Scavenging hydroxyl and DPPH radicals, inhibiting activities of *α*-amylase and *α*-glycosidase, and stimulating NO production | A polysaccharide with high contents of carbohydrate, uronic acid, and polyphenol, and low Mw had stronger antioxidant activity |  |
| 104 | EPS-C |  |  |  |  |
| 105 | EPS-D | Antioxidant activity | Scavenging DPPH and hydroxyl radicals and owning TEAC and FRAP | A polysaccharide with high contents of carbohydrate, uronic acid, and polyphenol had stronger antioxidant activity | Wang et al., 2019b |
| 106 | EPS-T |  |  |  |  |
| 107 | DPRG | Neuroprotective activity | Increasing permeability level of mitochondrial membrane and decreasing protein expression of cytochrome *c* | Biological activity of a polysaccharide was usually limited by high Mw | Yang et al., 2019 |
| 108 | Not given | Antioxidant and anti-tumor activities | Scavenging DPPH and superoxide radicals, and inhibiting proliferation of cancer cells | A polysaccharide extracted by ultrasound combined with microwave treatment exhibited higher biological activity | Ying et al., 2019 |
| 109 | PPB-2 | Hypoglycemic and hepatoprotective activities | Inhibiting Glc diffusion, activities of *α*-amylase, *α*-glucosidase, ALT, and AST, and levels of TC, TG, TB, and MDA, as well as increasing activities of SOD and CAT, and GSH level | Biological activity of a polysaccharide might depend on its Mw, configuration, chemical composition, and so on | Zhang et al., 2019 |
| 110 | PL-N1 | Hepatoprotective activity | Decreasing expression of cytochrome P450 2E1 and release of cytokines, as well as increasing levels of UGTs and SULTs | ND | Chen et al., 2020 |
| 111 | SSEPS2 | Anti-tumor activity | Inhibiting proliferation of cancer cells | ND | Cheng et al., 2020a |
| 112 | SSIPS1 | Hypoglycemic activity | Inhibiting activities of *α*-amylase and *α*-glucosidase, as well as increasing glucose metabolism in cancer cells and activities of hexokinase and pyruvate kinase | ND | Cheng et al., 2020b |
| 113 | Not given | Antioxidant and protective activities | Scavenging DPPH, superoxide, and hydroxyl radicals, and increasing mRNA expression of GCLC, NQO1, and GCLM in Nrf2 signaling pathway | ND | Hu et al., 2020 |
| 114 | SVP | Anti-tumor activity | Inducing apoptosis, regulating cell cycle, inhibiting migration and invasion, enhancing activation of p53-related genes, and down-regulating MMP expression | ND | Wan et al., 2020 |
| 115 | PL-N | Antioxidant and protective activities | Scavenging DPPH and hydroxyl radicals, improving viscera indices of aging mice, enhancing antioxidant enzymes activities and TEAC, as well as reducing MDA level | ND | Wang et al., 2020 |
| 116 | Not given | Anti-fatigue activity | Reducing lactic acid content and creatine kinase activity, repairing damaged muscle cells, as well as inhibiting hypoglycemia | ND | Zhong, 2020 |
| 117 | Not given | ND | ND | ND | Chang et al., 2021 |
| 118 | Not given |  |  |  |  |
| 119 | Not given |  |  |  |  |
| 120 | Not given |  |  |  |  |
| 121 | PSeP | Antioxidant, anti-inflammatory, and wound healing activities | Reducing levels of ROS and MDA, increasing activities of GSH-Px and CAT, as well as inhibiting MPO activity | ND | Luo et al., 2021 |
| 122 | SHPS-1 | Anti-inflammatory activity | Decreasing pro-inflammatory genes (iNOS and TNF-*α*), and increasing anti-inflammatory (IL-10) and tissue repairing (macrophage Man receptor CD 206) genes | Branched structure, high Gal content and enrichment of linkages →3)-*β*-_D_-Glc*p*-(1→ of a polysaccharide may contribute to anti-inflammatory activity. Man in the outside of side chains of SHPS-1 stimulate tissue repairing activity | Sun et al., 2021 |
| 123 | SePSP | Antioxidant and anti-inflammatory activities | Scavenging DPPH, hydroxyl, and superoxide radicals, exhibiting stronger reducing power, decreasing MDA concentration, downregulating levels of TNF-*α* and IL-1*β* mRNA, and upregulating level of IL-10 mRNA | ND | Zuo et al., 2021 |
| 124 | PLPS | Antioxidant activity | Scavenging hydroxyl and DPPH radicals and exhibiting stronger TEAC and FRAP | A polysaccharide with low-Mw fractions and higher amounts of O−H groups owned higher antioxidant activities | Wu et al., 2022 |
| 125 | C-PIPS |  |  |  |  |

***Abbreviations***: Mw, molecular weight; Fru, fructose; Gal, galactose; Glc, glucose; Glc*p*, glucopyranosyl; Man, mannose; Suc, sucrose; QR, quinone oxidoreductase; GST, glutathione S-transferase; NQO, NAD(P)H quinone oxidoreductase; GCLC, glutamate-cysteine ligase, catalytic subunit; GCLM, glutamate-cysteine ligase, modifier subunit; NO, nitric oxide; TNF, tumor necrosis factor; IFN, interferon; IL, interleukin; SOD, superoxide dismutase; CAT, catalase; GSH, glutathione; GSH-Px, glutathione peroxidase; TEAC, trolox equivalent antioxidant capacity; MDA, malondialdehyde; DPPH, 1,1-diphenyl-2-picrylhydrazyl; ABTS, 2,2′-azino-bis(3-ethylbenzothiazoline-6-sulfonic acid); ROS, reactive oxygen species; VCAM, vascular cell adhesion molecule; JNK, c-Jun N-terminal kinase; NF, nuclear factor; MyD, myeloid differentiation factor; TRIF, Toll/IL-1 receptor domain containing adaptor inducing IFN-*β*; MAPK, mitogen-activated protein kinase; FRAP, ferric reducing ability of plasma; VEGF, vascular endothelial growth factor; Nrf, nuclear factor E2-related factor; COX, cyclooxygenase; IP, IFN-inducible protein; ALT, alanine aminotransferase; AST, aspartate aminotransferase; TC, total cholesterol; TG, total triglycerides; TB, total bilirubin; UGT, glucuronosyltransferase; SULT, sulfotransferase; MMP, matrix metalloproteinase; MPO, myeloperoxidase; iNOS, inducible NO synthase; ND, not detected.

***References***

Baker, J. R., Kim, J. S., and Park, S. Y. (2008). Composition and proposed structure of a water-soluble glycan from the Keumsa Sangwhang Mushroom (*Phellinus linteus*). *Fitoterapia* 79, 345−350. doi: 10.1016/j.fitote.2008.03.002

Cao, C. L., Peng, F., and Cui, B. K. (2013). Chemical characterization and structure of exopolysaccharides from submerged culture of new medicinal mushroom from China, *Phellinus mori* (higher basidiomycetes). *Int. J. Med. Mushrooms* 15, 57−69. doi: 10.1615/IntJMedMushr.v15.i1.70

Chang, C., Zhao, J. H., Yu, W. J., Chen, Q. H., Qin, L. W., Wu, X. L., et al. (2021). Extraction technology and determination of polysaccharide from *Sanghuangporus vaninii*. *Chem. Reag.* 43, 973−978. doi: 10.13822 /j.cnki.hxsj.2021007987

Chen, C., Liu, X., Qi, S. S., Dias, A. C. P., Yan, J. K., and Zhang, X. Y. (2020). Hepatoprotective effect of *Phellinus linteus* mycelia polysaccharide (PL-N1) against acetaminophen-induced liver injury in mouse. *Int. J. Biol. Macromol.* 154, 1276−1284. doi: 10.1016/j.ijbiomac.2019.11.002

Chen, L., Pan, J. Z., Li, X., Zhou, Y., Meng, Q. L., and Wang, Q. (2011). Endo-polysaccharide of *Phellinus igniarius* exhibited anti-tumor effect through enhancement of cell mediated immunity. *Int. Immunopharmacol.* 11, 255−259. doi: 10.1016/j.intimp.2010.11.033

Cheng, J. W., Song, J. L., Liu, Y., Lu, N., Wang, Y. B., Hu, C. J., et al. (2020a). Conformational properties and biological activities of *α*-_D_-mannan from *Sanghuangporus sanghuang* in liquid culture. *Int. J. Biol. Macromol.* 164, 3568−3579. doi: 10.1016/j.ijbiomac.2020.08.112

Cheng, J. W., Song, J. L., Wei, H. L., Wang, Y. B., Huang, X. B., Liu, Y., et al. (2020b). Structural characterization and hypoglycemic activity of an intracellular polysaccharide from *Sanghuangporus sanghuang* mycelia. *Int. J. Biol. Macromol.* 164, 3305−3314. doi: 10.1016/j.ijbiomac.2020.08.202

Cheng, W., Qin, J. Z., Du, J. G., and Zhang, C. H. (2012). Optimization of ultrasonic-assisted enzymatic extraction of polysaccharide from *Phellinus igniarius*. *Mod. Food Sci. Technol.* 28, 662−666. doi: 10.3390/molecules24010147

Gao, W. W., Wang, W. D., Sun, W. J., Wang, M. F., Zhang, N., and Yu, S. W. (2017). Antitumor and immunomodulating activities of six *Phellinus igniarius* polysaccharides of different origins. *Exp. Ther. Med.* 14, 4627−4632. doi: 10.3892/etm.2017.5191

Ge, Q., Mao, J. W., Zhang, A. Q., Wang, Y. J., and Sun, P. L. (2013). Purification, chemical characterization, and antioxidant activity of a polysaccharide from the fruiting bodies of sanghuang mushroom (*Phellinus baumii* Pilát). *Food Sci. Biotechnol.* 22, 301−307. doi: 10.1007/s10068-013-0081-1

Ge, Q., Zhang, A. Q., and Sun, P. L. (2009a). Purification and structural elucidation of a novel fucoglucan from the fruiting bodies of *Phellinus baumii* Pilát. *J. Sci. Food Agr.* 89, 343−348. doi: 10.1002/jsfa.3464

Ge, Q., Zhang, A. Q., and Sun, P. L. (2009b). Structural investigation of a novel water-soluble heteropolysaccharide from the fruiting bodies of *Phellinus baumii* Pilát. *Food Chem.* 114, 391−395. doi: 10.1016/j.foodchem.2008.09.010

Guo, X., Zou, X., and Sun, M. (2010). Optimization of extraction process by response surface methodology and preliminary characterization of polysaccharides from *Phellinus igniarius*. *Carbohyd. Polym.* 80, 344−349. doi: 10.1016/j.carbpol.2009.11.028

He, P. X., Geng, L. J., Wang, J. Z., Wang, Z., Mao, D. B., and Xu, C. P. (2012). Purification, characterization and bioactivity of an extracellular polysaccharide produced from *Phellinus igniarius*. *Ann. Microbiol.* 62, 1697−1707. doi: 10.1007/s13213-012-0427-6

Hu, X. T., Ye, Y. J., Shi, G., Zhao, N. X., Gu, M. L., Yan, Y. N., et al. (2020). Extraction of polysaccharides from fruiting bodies of *Phellinus igniarius* and its protective effect on _D_-galactose induced 3T3 cell injury. *Food Sci.* 41, 204−211. doi: 10.7506/spkx1002-6630-20190906-078

Hwang, H. J., Kim, S. W., Choi, J. W., and Yun, J. W. (2003). Production and characterization of exopolysaccharides from submerged culture of *Phellinus linteus* KCTC 6190. *Enzyme Microb. Technol.* 33, 309−319. doi: 10.1016/S0141-0229(03)00131-5

Jia, X. W., Gao, M. Q., Li, M. Z., Wu, Y., Zeng, Y., and Xu, C. P. (2017). Molecular characterization of two polysaccharides from *Phellinus vaninii* Ljup and their cytotoxicity to cancer cell lines. *Anti-Cancer Age. Med. Chem.* 17, 1−8. doi: 10.2174/1871520617666170912144956

Jiang, P., Yuan, L., Cai, D. L., Jiao, L. L., and Zhang, L. P. (2015). Characterization and antioxidant activities of the polysaccharides from mycelium of *Phellinus pini* and culture medium. *Carbohyd. Polym.* 117, 600−604. doi: 10.1016/j.carbpol.2014.10.013

Jiang, P., Yuan, L., Huang, G. H., Wang, X. L., Li, X., Jiao, L. L., et al. (2016). Structural properties and immunoenhancement of an exopolysaccharide produced by *Phellinus pini*. *Int. J. Biol. Macromol.* 93, 566−571. doi: 10.1016/j.ijbiomac.2016.09.020

Jin, Q. L., Zhang, Z. F., Lv, G. Y., Cai, W. M., Cheng, J. W., Wang, J. G., et al. (2016). Antioxidant and DNA damage protecting potentials of polysaccharide extracted from *Phellinus baumii* using a delignification method. *Carbohyd. Polym.* 152, 575−582. doi: 10.1016/j.carbpol.2016.07.027

Kim, G. Y., Lee, J. Y., Lee, J. O., Ryu, C. H., Choi, B. T., Jeong, Y. K., et al. (2006). Partial characterization and immunostimulatory effect of a novel polysaccharide-protein complex extracted from *Phellinus linteus*. *Biosci. Biotechnol. Biochem.* 70, 1218−1226. doi: 10.1271/bbb.70.1218

Kim, G. Y., Park, H. S., Nam, B. H., Lee, S. J., and Lee, J. D. (2003). Purification and characterization of acidic proteo-heteroglycan from the fruiting body of *Phellinus linteus* (Berk. & M.A. Curtis) Teng. *Bioresour. Technol.* 89, 81−87. doi: 10.1016/S0960-8524(02)00273-0

Kim, H. M., Kang, J. S., Kim, J. Y., Park, S. K., Kim, H. S., Lee, Y. J., et al. (2010). Evaluation of antidiabetic activity of polysaccharide isolated from *Phellinus linteus* in non-obese diabetic mouse. *Int. Immunopharmacol.* 10, 72−78. doi: 10.1016/j.intimp.2009.09.024

Lee, S. M., Kim, S. M., Lee, Y. H., Kim, W. J., Park, J. K., Park, Y. I., et al. (2010). Macromolecules isolated from *Phellinus pini* fruiting body: Chemical characterization and antiviral activity. *Macromol. Res.* 18, 602−609. doi: 10.1007/s13233-010-0615-9

Li, R. X., Wang, Y. T., Xia, J. F., Luo, D. Q., and Wang, T. C. (2019). Optimization of extraction process of polysaccharide of *Phellinus igniarius* mycelium and analysis of its antioxidant activity *in vitro*. *Chin. Agr. Sci. Bull.* 35, 143−150.

Li, S. C., Yang, X. M., Ma, H. L., Yan, J. K., and Guo, D. Z. (2015). Purification, characterization and antitumor activity of polysaccharides extracted from *Phellinus igniarius* mycelia. *Carbohyd. Polym.* 133, 24−30. doi: 10.1016/j.carbpol.2015.07.013

Li, T. T., Yang, Y., Liu, Y. F., Zhou, S., Yan, M. Q., Wu, D., et al. (2015). Physicochemical characteristics and biological activities of polysaccharide fractions from *Phellinus baumii* cultured with different methods. *Int. J. Biol. Macromol.* 81, 1082−1088. doi: 10.1016/j.ijbiomac.2015.09.001

Li, X., Jiao, L. L., Zhang, X., Tian, W. M., Chen, S., and Zhang, L. P. (2008). Anti-tumor and immunomodulating activities of proteoglycans from mycelium of *Phellinus nigricans* and culture medium. *Int. Immunopharmacol.* 8, 909−915. doi: 10.1016/j.intimp.2008.02.008

Liu, M. M., Zeng, P., Li, X. T., and Shi, L. G. (2016). Antitumor and immunomodulation activities of polysaccharide from *Phellinus baumii*. *Int. J. Biol. Macromol.* 91, 1199−1205. doi: 10.1016/j.ijbiomac.2016.06.086

Liu, X., Hou, R. L., Xu, K. Q., Chen, L., Wu, X. P., Lin, W. X., et al. (2019a). Extraction, characterization and antioxidant activity analysis of the polysaccharide from the solid-state fermentation substrate of *Inonotus hispidus*. *Int. J. Biol. Macromol.* 123, 468−476. doi: 10.1016/j.ijbiomac.2018.11.069

Liu, X., Hou, R. L., Yan, J. J., Xu, K. Q., Wu, X. P., Lin, W. X., et al. (2019b). Purification and characterization of *Inonotus hispidus* exopolysaccharide and its protective effect on acute alcoholic liver injury in mice. *Int. J. Biol. Macromol.* 129, 41−49. doi: 10.1016/j.ijbiomac.2019.02.011

Liu, Y. H., Liu, C. H., Jiang, H. Q., Zhou, H. L., Li, P. L., and Wang, F. S. (2015). Isolation, structural characterization and neurotrophic activity of a polysaccharide from *Phellinus ribis*. *Carbohyd. Polym.* 127, 145−151. doi: 10.1016/j.carbpol.2015.03.057

Liu, Y. H., and Wang, F. S. (2007). Structural characterization of an active polysaccharide from *Phellinus ribis*. *Carbohyd. Polym.* 70, 386−392. doi: 10.1016/j.carbpol.2007.04.019

Liu, Y. H., Xu, J. Z., Zong, A. Z., Wang, J. H., Liu, Y. G., Jia, W., et al. (2018). Anti-angiogenic activity and mechanism of a chemically sulfated natural glucan from *Phellinus ribis*. *Int. J. Biol. Macromol.* 107, 2475−2483. doi: 10.1016/j.ijbiomac.2017.10.134

Luo, J. G., Liu, J., Ke, C. L., Qiao, D. L., Ye, H., Sun, Y., et al. (2009). Optimization of medium composition for the production of exopolysaccharides from *Phellinus baumii* Pilát in submerged culture and the immuno-stimulating activity of exopolysaccharides. *Carbohyd. Polym.* 78, 409−415. doi: 10.1016/j.carbpol.2009.04.038

Luo, J. G., Liu, J., Sun, Y., Ye, H., Zhou, C. H., and Zeng, X. X. (2010). Medium optimization, preliminary characterization and antioxidant activity *in vivo* of mycelial polysaccharide from *Phellinus baumii* Pilát. *Carbohyd. Polym.* 81, 533−540. doi: 10.1016/j.carbpol.2010.03.010

Luo, L. J., Wang, Y. X., Zhang, S., Guo, L., Jia, G. T., Lin, W. P., et al. (2021). Preparation and characterization of selenium-rich polysaccharide from *Phellinus igniarius* and its effects on wound healing. *Carbohyd. Polym.* 264, 117982. doi: 10.1016/j.carbpol.2021.117982

Ma, X. K., Guo, D. D., Peterson, E. C., Dun, Y., and Li, D. Y. (2016). Structural characterization and anti-aging activity of a novel extracellular polysaccharide from fungus *Phellinus* sp. in mammalian system. *Food Funct.* 7, 3468−3479. doi: 10.1039/C6FO00422A

Ma, X. K., She, X., Peterson, E. C., Wang, Y. Z., Zheng, P., Ma, H. Y., et al. (2019). A newly characterized exopolysaccharide from *Sanghuangporus sanghuang*. *Int. J. Biol. Macromol.* 57, 812−820. doi: 10.1007/s12275-019-9036-4

Mei, Y. X., Zhu, H., Hu, Q. M., Liu, Y. Y., Zhao, S. M., Peng, N., et al. (2015). A novel polysaccharide from mycelia of cultured *Phellinus linteus* displays antitumor activity through apoptosis. *Carbohyd. Polym.* 124, 90−97. doi: 10.1016/j.carbpol.2015.02.009

Pei, J. J., Wang, Z. B., Ma, H. L., and Yan, J. K. (2015). Structural features and antitumor activity of a novel polysaccharide from alkaline extract of *Phellinus linteus* mycelia. *Carbohyd. Polym.* 115, 472−477. doi: 10.1016/j.carbpol.2014.09.017

Shi, Y. B., Bai, W. D., Zhao, W. H., Qian, M., and Bai, Y. L. (2019). Optimization of enzymatic hydrolysis-assisted extraction of polysaccharides from *Phellinus igniarius*. *Farm Prod. Process.* 9, 29−32+35. doi: 10.16693/j.cnki.1671-9646(X).2019.09.042

Shon, Y. H., and Nam, K. S. (2002). Cancer chemoprevention: Inhibitory effect of soybeans fermented with basidiomycetes on 7,12-dimethylbenz[a]anthracene/12-*O*-tetradecanoylphorbol-13-acetate-induced mouse skin carcinogenesis. *Biotechnol. Lett.* 24, 1005−1010.

Shon, Y. H., and Nam, K. S. (2003). Inhibition of cytochrome P450 isozymes in rat liver microsomes by polysaccharides derived from *Phellinus linteus*. *Biotechnol. Lett.* 25, 167−172. doi: 10.1016/S0308-8146(03)00015-3

Suabjakyong, P., Nishimura, K., Toida, T., and van Griensven, L. J. L. D. (2015). Structural characterization and immunomodulatory effects of polysaccharides from *Phellinus linteus* and *Phellinus igniarius* on the IL-6/IL-10 cytokine balance of the mouse macrophage cell lines (RAW264.7). *Food Funct.* 6, 2834−2844. doi: 10.1039/c5fo00491h

Sun, Y. Q., Huo, J. X., Zhong, S., Zhu, J. X., Li, Y. G., and Li, X. J. (2021). Chemical structure and anti-inflammatory activity of a branched polysaccharide isolated from *Phellinus baumii*. *Carbohyd. Polym.* 268, 118214. doi: 10.1016/j.carbpol.2021.118214

van Griensven, L. J. L. D., and Verhoeven, H. A. (2013). *Phellinus linteus* polysaccharide extracts increase the mitochondrial membrane potential and cause apoptotic death of THP-1 monocytes. *Chin. Med.* 8, 25. doi: 10.1186/1749-8546-8-25

Wan, X. L., Jin, X., Xie, M. L., Liu, J., Gontcharov, A. A., Wang, H., et al. (2020). Characterization of a polysaccharide from *Sanghuangporus vaninii* and its antitumor regulation via activation of the p53 signaling pathway in breast cancer MCF-7 cells. *Int. J. Biol. Macromol.* 163, 865−877. doi: 10.1016/j.ijbiomac.2020.06.279

Wang, K., Ding, Z. C., Pei, J. J., and Yan, J. K. (2020). Antioxidant activities of polysaccharides from *Phellinus linteus* mycelia by alkaline extraction. *Sci. Technol. Food Ind.* 41, 289−294. doi: 10.13386/j.issn1002-0306.2020.01.047

Wang, L., Yao, L., Jin, Y. W., Jin, C. Y., Dong, Y., Shou, D., et al. (2019). Activation effect of human TLR4 signaling pathway by polysaccharide from *Phellinus igniarius*. *Chin. J. Mod. Appl. Pharm.* 36, 1178−1182. doi: 10.13748/j.cnki.issn1007-7693.2019.10.002

Wang, Y. Q., Mao, J. B., Zhou, M. Q., Jin, Y. W., Lou, C. H., Dong, Y., et al. (2019). Polysaccharide from *Phellinus igniarius* activates TLR4-mediated signaling pathways in macrophages and shows immune adjuvant activity in mice. *Int. J. Biol. Macromol.* 123, 157−166. doi: 10.1016/j.ijbiomac.2018.11.066

Wang, Y. Y., Ma, H. L., Ding, Z. C., Yang, Y., Wang, W. H., Zhang, H. N., et al. (2019a). Three-phase partitioning for the direct extraction and separation of bioactive exopolysaccharides from the cultured broth of *Phellinus baumii*. *Int. J. Biol. Macromol.* 123, 201−209. doi: 10.1016/j.ijbiomac.2018.11.065

Wang, Y. Y., Ma, H. L., Yan, J. K., Wang, K. D., Yang, Y., Wang, W. H., et al. (2019b). Three-phase partitioning system with dimethyl carbonate as organic phase for partitioning of exopolysaccharides from *Phellinus baumii*. *Int. J. Biol. Macromol.* 131, 941−948. doi: 10.1016/j.ijbiomac.2019.03.149

Wang, Z. B., Pei, J. J., Ma, H. L., Cai, P. F., and Yan, J. K. (2014). Effect of extraction media on preliminary characterizations and antioxidant activities of *Phellinus linteus* polysaccharides. *Carbohyd. Polym.* 109, 49−55. doi: 10.1016/j.carbpol.2014.03.057

Wang, Z. Y., Wang, C. Y., and Quan, Y. (2014a). Extraction of polysaccharides from *Phellinus nigricans* mycelia and their antioxidant activities *in vitro*. *Carbohyd. Polym.* 99, 110−115. doi: 10.1016/j.carbpol.2013.08.073

Wang, Z. Y., Zhou, F., and Quan, Y. (2014b). Antioxidant and immunological activity *in vitro* of polysaccharides from *Phellinus nigricans* mycelia. *Int. J. Biol. Macromol.* 64, 139−143. doi: 10.1016/j.ijbiomac.2013.11.038

Wu, S. J., Liaw, C. C., Pan, S. Z., Yang, H. C., and Ng, L. T. (2013). *Phellinus linteus* polysaccharides and their immunomodulatory properties in human monocytic cells. *J. Funct. Foods* 5, 679−688. doi: 10.1016/j.jff.2013.01.011

Wu, Y., Liu, H., Li, Z. H., Huang, D. Y., Nong, L. Z., Ning, Z. X., et al. (2022). Purification of polysaccharides from *Phellinus linteus* by using an aqueous two-phase system and evaluation of the physicochemical and antioxidant properties of polysaccharides *in vitro*. *Prep. Biochem. Biotechnol.* 52, 89−98. doi: 10.1080/10826068.2021.1911815

Xu, C. P., Yu, J. W., Zhao, S. S., Wu, S. S., He, P. X., Jia, X. W., et al. (2017). Effect of carbon source on production, characterization and bioactivity of exopolysaccharide produced by *Phellinus vaninii* Ljup. *An. Acad. Bras. Ciênc.* 89, 2033−2041. doi: 10.1590/0001-3765201720150786

Xu, Y., Zhao, X. Y., Cao, H., Sheng, S., Wang, J., and Wu, F. A. (2016). Enzyme-catalyzed extraction and antioxidant activity of polysaccharides from *Phellinus igniarius*. *Curr. Top. Nutraceut. Res.* 14, 171−180.

Xue, Q., Sun, J., Zhao, M. W., Zhang, K. Y., and Lai, R. (2011). Immunostimulatory and anti-tumor activity of a water-soluble polysaccharide from *Phellinus baumii* mycelia. *World J. Microbiol. Biotechnol.* 27, 1017−1023. doi: 10.1007/s11274-010-0545-x

Yan, J. K., Wang, Y. Y., Ma, H. L., and Wang, Z. B. (2016a). Ultrasonic effects on the degradation kinetics, preliminary characterization and antioxidant activities of polysaccharides from *Phellinus linteus* mycelia. *Ultrason. Sonochem.* 29, 251−257. doi: 10.1016/j.ultsonch.2015.10.005

Yan, J. K., Wang, Y. Y., Ma, H. L., Wang, Z. B., and Pei, J. J. (2016b). Structural characteristics and antioxidant activity *in vivo* of a polysaccharide isolated from *Phellinus linteus* mycelia. *J. Taiwan Inst. Chem. Eng.* 65, 110−117. doi: 10.1016/j.jtice.2016.05.052

Yan, J. K., Wang, Y. Y., Wang, Z. B., Ma, H. L., Pei, J. J., and Wu, J. Y. (2016c). Structure and antioxidative property of a polysaccharide from an ammonium oxalate extract of *Phellinus linteus*. *Int. J. Biol. Macromol.* 91, 92−99. doi: 10.1016/j.ijbiomac.2016.05.063

Yang, P., Jin, J., Liu, Q., Ma, D. M., Li, J., Zhang, Y. Q., et al. (2019). Optimization of degradation conditions with PRG, a polysaccharide from *Phellinus ribis*, by RSM and the neuroprotective activity in PC12 cells damaged by A*β*_25-35_. *Molecules* 24, 3010. doi: 10.3390/molecules24163010

Yang, Y., Ye, L. B., Zhang, J. S., Liu, Y. F., and Tang, Q. J. (2009). Structural analysis of a bioactive polysaccharide, PISP1, from the medicinal mushroom *Phellinus igniarius*. *Biosci. Biotechnol. Biochem.* 73, 134−139. doi: 10.1271/bbb.80546

Yang, Y., Zhang, J. S., Liu, Y. F., Tang, Q. J., Zhao, Z. G., and Xia, W. S. (2007). Structural elucidation of a 3-*O*-methyl-_D_-galactose-containing neutral polysaccharide from the fruiting bodies of *Phellinus igniarius*. *Carbohyd. Res.* 342, 1063−1070. doi: 10.1016/j.carres.2007.02.019

Ying, R. F., Huang, M. G., Wang, Y. S., Wu, C. E., Li, T. T., and Fan, G. J. (2019). Ultrasonic-microwave synergistic assisted extraction and activity of polysaccharides from *Phellinus igniarius*. *Food Res. Dev.* 40, 82−88. doi: 10.12161/j.issn.1005-6521.2019.21.015

Ying, R. F., Wu, C. E., Huang, M. G., and Wang, Y. S. (2017). Anti-tumor activity of polysaccharides from *Phellinus igniarius* fruiting body and mycelium. *China Food Addit.* 12, 57−61.

Yuan, Q. X., Zhao, L. Y., Li, Z. H., Harqin, C., Peng, Y. F., and Liu, J. K. (2018). Physicochemical analysis, structural elucidation and bioactivities of a high-molecular-weight polysaccharide from *Phellinus igniarius* mycelia. *Int. J. Biol. Macromol.* 120, 1855−1864. doi: 10.1016/j.ijbiomac.2018.09.192

Zhang, H. N., Ma, H. L., Liu, W., Pei, J. J., Wang, Z. B., Zhou, H. J., et al. (2014). Ultrasound enhanced production and antioxidant activity of polysaccharides from mycelial fermentation of *Phellinus igniarius*. *Carbohyd. Polym.* 113, 380−387. doi: 10.1016/j.carbpol.2014.07.027

Zhang, H. N., Ma, H. L., Zhou, C. S., Yan, Y., Yin, X. L., and Yan, J. K. (2018). Enhanced production and antioxidant activity of endo-polysaccharides from *Phellinus igniarius* mutants screened by low power He-Ne laser and ultraviolet induction. *Bioact. Carbohyd. Diet. Fibre* 15, 30−36. doi: 10.1016/j.bcdf.2016.11.006

Zhang, Z. F., Lv, G. Y., Cheng, J. W., Cai, W. M., Fan, L. F., and Miao, L. X. (2019). Characterization and biological activities of polysaccharides from artificially cultivated *Phellinus baumii*. *Int. J. Biol. Macromol.* 129, 861−868. doi: 10.1016/j.ijbiomac.2019.02.082

Zhang, Z. F., Lv, G. Y., Song, T. T., Jin, Q. L., Huang, J. B., Fan, L. F., et al. (2015). Comparison of the preliminary characterizations and antioxidant properties of polysaccharides obtained from *Phellinus baumii* growth on different culture substrates. *Carbohyd. Polym.* 132, 397−399. doi: 10.1016/j.carbpol.2015.06.006

Zhao, C., Liao, Z. S., Wu, X. Q., Liu, Y. L., Liu, X. Y., Lin, Z. X., et al. (2014). Isolation, purification, and structural features of a polysaccharide from *Phellinus linteus* and its hypoglycemic effect in alloxan-induced diabetic mice. *J. Food Sci.* 79, H1002−H1010. doi: 10.1111/1750-3841.12464

Zhong, C. (2020). Anti-fatigue effect of fermentation broth polysaccharose of *Phellinus igniarius*. *Edible Fungi China* 39, 46−48. doi: 10.13629/j.cnki.53-1054.2020.06.012

Zuo, K., Tang, K. J., Liang, Y., Xu, Y. F., Sheng, K. L., Kong, X. W., et al. (2021). Purification and antioxidant and anti-inflammatory activity of extracellular polysaccharopeptide from sanghuang mushroom, *Sanghuangporus lonicericola*. *J. Sci. Food Agr.* 101, 1009−1020. doi: 10.1002/jsfa.10709
